# Aramid Fiber-Reinforced Plastics (AFRPs) in Aerospace: A Review of Recent Advancements and Future Perspectives

**DOI:** 10.3390/polym17162254

**Published:** 2025-08-20

**Authors:** Xinning Xu, Yanbing Guo, Zhikang Shen, Boyang Liu, Fei Yan, Ning Zhong

**Affiliations:** 1 Institute of Marine Materials Science and Engineering, College of Ocean Science and Engineering, Shanghai Maritime University, Shanghai 201306, China; 2 College of Engineering and Technology, Southwest University, Chongqing 400715, China; 3 School of Automobile Engineering, Wuhan University of Technology, Wuhan 430070, China

**Keywords:** aramid fiber reinforced plastics, aerospace structural applications, interface engineering, advanced manufacturing technologies, structure–property relationship

## Abstract

This review examines the application of aramid fiber-reinforced plastics (AFRPs) in the aerospace industry, highlighting their significance in enhancing aircraft performance. Aramid fibers, such as Kevlar^®^ and Twaron^®^, have emerged as key materials due to their exceptional tensile strength, low density, and thermal stability. However, challenges persist in manufacturing, durability, and multifunctionality. This paper evaluates the latest advancements in AFRP, focusing on how molecular structure, interfacial engineering, and manufacturing innovations influence performance. It addresses questions on improving adhesion, efficient manufacturing methods, enhancing durability under extreme conditions, and developing multifunctional AFRP. By analyzing breakthroughs from 2020 to 2025 and proposing targeted solutions, this review aims to help AFRP meet the demands of future aerospace systems.

## 1. Introduction

The aerospace industry’s relentless pursuit of enhanced performance metrics, including weight reduction, strength improvement, and superior environmental adaptability, has drawn significant attention to advanced composite materials [1]. Since the Wright brothers’ first aircraft, “Flyer I,” in 1903, aircraft materials have evolved from wood and fabric structures (prone to decay and high maintenance) to all-metal designs using steel tubes and aluminum plates by the 1930s. As aviation demands grew, the need for lighter structures led to the adoption of composite materials. Glass fiber-reinforced plastic (GFRP) composites were first used in aircraft cowlings, noses, and cockpits in the 1940s. By the 1970s, with the invention of Kevlar^®^ (aramid) fibers and carbon fibers by DuPont, composites gradually expanded their role in aircraft structures. Today, modern aircraft, like the Boeing 787 and Airbus A350, utilize over 50% composite parts, with the 787 using composites for approximately 50% of its weight—a significant increase from the 777’s 12% composites and 50% aluminum.

Among advanced composites, aramid fiber-reinforced composites (AFRPs) stand out for their unique combination of properties. However, the value of AFRP does not derive from its absolute leadership in any single metric but instead from its unique ‘performance ecosystem’—that is, applications where toughness, impact resistance, and environmental durability are valued more highly than absolute stiffness or strength. This positioning stems from a series of inherent trade-offs in performance. This trade-off is further evident when comparing AFRP with other fiber-reinforced plastics, such as CFRP and GFRP, across mechanical, thermal, and economic characteristics, as integrated from hybrid composite studies. Mechanically, AFRP offers high tensile strength (3.0–3.6 GPa) and superior impact resistance, with the Charpy impact strength in CFRTP/AFRTP hybrids reaching 63–80 kJ/m^2^—comparable to pure AFRTP (65 kJ/m^2^) and 2–3 times higher than CFRTP (≈24 kJ/m^2^)—making it ideal for impact-prone aerospace structures, like aircraft fuselages. CFRP excels in stiffness with a specific modulus of 200–350 GPa/g/cm^3^ (2.5–4 times that of AFRP at 85–120 GPa/g/cm^3^) but shows brittle failure under impact, and GFRP provides moderate flexural strength (around 400–500 MPa in hybrids) with lower modulus (35–50 GPa/g/cm^3^) and good anti-corrosion properties but vulnerability to creep and alkaline degradation [2,3,4]. Thermally, AFRP demonstrates stability with a glass transition temperature (Tg) of 135–160 °C under 4 MPa and 90 s molding, closely matching CFRP’s Tg of 160 °C, yet after thermal exposure at 200 °C, AFRP experiences a ≈35% drop in compressive strength versus ≈45% for CFRP and ≈50–60% for GFRP, highlighting its moderate advantage in residual properties for high-temperature aerospace environments, though all suffer from matrix degradation and potential delamination [3,5,6]. Economically, AFRP’s cost (80–120 USD/kg) offers efficiency in hybrids (77.3% normalized to CFRP), becoming preferable for regional jets when fuel prices are below 1.5 USD/gallon per variable fuel-price models, whereas CFRP (120–250 USD/kg) gains competitiveness at higher fuel costs for long-range aircraft, and GFRP (25–45 USD/kg, 13.3% normalized to CFRP) provides the highest cost/strength ratio for low-demand applications [4,7]. These comparisons are consolidated in Table 1 for clarity.

Lockheed Martin’s F-35 Joint Strike Fighter is a case in point: its spacer structure is made of three-dimensional woven AFRP, which not only achieves a weight reduction of 23 percent compared to titanium alloys, but also demonstrates a ballistic impact resistance 1.8 times higher than that of carbon fiber composites in the NATO STANAG 2920 test [9]. This indicates that the value of AFRP does not come from simply being ‘light’, but from the combination of ‘light and tough’ properties. In space applications, AFRP composites are a key component of thermal protection systems that protect spacecraft structures during re-entry [10].

Recent advances in molecular characterization have revealed that commercial para-aramids exhibit 68–72% crystallinity, compared to 45–50% in experimental meta-aramid hybrids, as demonstrated by synchrotron WAXS studies [11,12]. Transmission electron microscopy (TEM) analyses further highlight how β-sheet alignment variations in para-aramid fibers directly influence compressive strength, with misaligned domains reducing axial load-bearing capacity by 12–15% [13,14].

However, despite these successes, the full potential of AFRP is fundamentally constrained by a series of interconnected challenges rooted in the material’s inherent properties and its interaction with the service environment. Environmental adaptability remains a critical barrier; recent studies confirm that under combined UV and thermal cycling (−55 °C to +150 °C), a 12–15% loss in compressive strength can be expected, while long-term humidity exposure (e.g., 85% RH for 1000 h) can degrade interlaminar shear strength by over 20% due to moisture attacking the fiber–matrix interface [15]. This vulnerability is exacerbated by the intrinsically weak interfacial bonding between the chemically inert aramid fiber surface and most polymer matrices, which leads to inefficient stress transfer and a predisposition to delamination under load [16,17]. Perhaps the most significant limitation is the material’s notoriously low compressive strength, often just 15–20% of its tensile capability, which severely restricts its use in primary load-bearing structures dominated by compressive forces [18]. Compounding these technical issues are high manufacturing costs and significant machining difficulties, which have historically limited their wider adoption beyond specialized applications [19,20].

To bridge the gap between the promise of AFRP and the demanding reality of next-generation aerospace systems, a multi-faceted approach is required. This critical review aims to synthesize and evaluate the latest advancements that address these core challenges. The landscape of recent breakthroughs (2020–2025) will be navigated by first establishing the fundamental principles of AFRP and then critically analyzing the key strategies for performance enhancement, including interfacial engineering, durability improvement, and the integration of multifunctionality. Subsequently, this review will explore how advanced manufacturing and assembly technologies are revolutionizing the production of complex AFRP components, making them more cost-effective and reliable. By examining insightful case studies and culminating in a forward-looking technology roadmap, this review provides a comprehensive framework for understanding and overcoming the existing hurdles, ultimately paving the way for the expanded and intelligent application of AFRP in future aerospace systems.

## 2. Fundamentals of Aramid Fibers and AFRP

To fully appreciate both the immense potential and the inherent limitations of AFRP in aerospace, it is essential first to understand the fundamental characteristics of its constituent materials. This section establishes the crucial link between the molecular structure of aramid fibers, the properties of polymer matrices, and the critical nature of their interface, which collectively govern the performance and challenges of the final composite.

### 2.1. Aramid Fibers: Classification, Synthesis, and Microstructure

**Figure 1:**
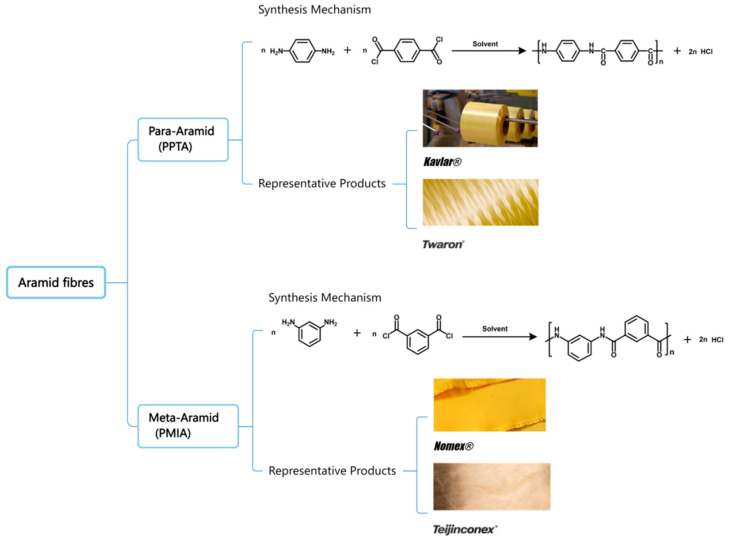
Classification of aramid fibers.

The US Federal Trade Commission defines aramid fibers (AFs) as “a manufactured fiber in which the fiber forming substance is a long-chain synthetic polyamide in which at least 85% of the amide bonds (-CO-NH-) are directly attached to two aromatic rings” [21]. This aromatic backbone is the source of their signature high performance. The synthesis is typically achieved through a low-temperature polycondensation reaction between aromatic diacyl chlorides and aromatic diamines in polar amide solvents. This method enables the formation of high-molecular-weight polymers, which are essential for producing high-strength fibers [20,22,23]. Based on the attachment points of the amide linkages on the benzene rings, aramid fibers are primarily categorized into two classes, para-aramids and meta-aramids, whose distinct molecular structures lead to vastly different properties and, consequently, particular roles in aerospace applications (as illustrated in Figure 1). Para-aramids (p-AF), such as DuPont’s Kevlar^®^ and Teijin’s Twaron^®^, are characterized by their rigid, linear, and highly oriented molecular chains (Figure 1). This rod-like structure, reinforced by extensive intermolecular hydrogen bonding, is the origin of their exceptionally high tensile strength (up to 3.6 GPa) and modulus, making them the primary choice for structural applications that demand high integrity and impact resistance [24,25]. In aerospace, this translates to the use of fuselages, wing components, and engine containment rings that must withstand blade-out events, as well as crucial ballistic protection layers [26].

As shown in Table 2, the properties of para-aramid (p-AF) and meta-aramid (m-AF) fibers differ, leading to their distinct specific applications in the aerospace field. Table 3 selects Kevlar^®^ and Nomex^®^ as the representative para-aramid (p-AF) and meta-aramid (m-AF), respectively, and lists their applications in the aerospace field. The chemical inertness of their surfaces (Table 2) and weak interfacial bonding with polymer matrices pose significant challenges for composite applications, necessitating the surface modification strategies discussed in Section 3.1.

In contrast, meta-aramids (m-AF), such as Nomex^®^ and Teijinconex^®^, feature a kinked or “zigzag” molecular chain structure (Figure 1). This configuration disrupts the tight molecular packing, resulting in lower mechanical strength but outstanding thermal stability, with a glass transition temperature (Tg) of around 270 °C, and inherent flame retardancy [24,25]. Consequently, their role in aerospace is predominantly in non-structural applications where heat and fire resistance are paramount. This includes thermal insulation blankets in engine compartments, fire-blocking layers in aircraft seats, and lightweight, flame-retardant honeycomb cores used in cabin flooring and interior panels [27].

The microstructure of para-aramid fibers is the fundamental source of both their greatest strengths and most significant weaknesses. X-ray diffraction studies reveal a highly crystalline and ordered structure, with rigid molecular chains aligned along the fiber axis [28]. This highly ordered, anisotropic microstructure, with strong covalent bonds along the fiber axis and weaker hydrogen bonds providing lateral cohesion, directly explains the material’s performance dichotomy: remarkable axial tensile strength and stiffness, but critically poor compressive strength (due to microbuckling of the rigid rods under compression) and low transverse and shear properties [29]. This inherent anisotropy is a central theme that dictates the design, manufacturing, and application challenges discussed throughout this review. Furthermore, the chemically inert and smooth surface of these fibers presents a significant challenge for achieving strong adhesion to polymer matrices. This critical point will be explored in Section 2.3 and addressed with solutions in Section 3.

**Table 3:** Applications of DuPont™ Kevlar^®^ and Nomex^®^ in the aerospace field [30].

Application Direction	Material	Advantages
Aircraft cabin flooring and interiors	Nomex^®^ or Kevlar^®^ honeycomb cores	Weight savings for aircraft manufacturers. Very low electrical conductivity and high fire resistance; Contribute to addressing the safety standards required by the industry; Superior thermal and sound insulation.
NE.Cms_InsertLanding gear doors	Nomex^®^ or Kevlar^®^ honeycombs	Strong and light, allowing for more efficient aircraft
Wing boxes and control surfaces	Nomex^®^ or Kevlar^®^ honeycombs	Lightweight; Lack of galvanic corrosion; Superior strength; Better than heavier, weaker, and corrosion-susceptible aluminum cores.
Filament-wound pressure bottles	Kevlar^®^ filament	Reduce the overall weight of the aircraft.
Engine nacelles	Nomex^®^ or Kevlar^®^ honeycomb core structures	Far stronger and lighter than earlier designs with an aluminum core.
Engine containment rings	Kevlar^®^ fabric	Help catch errant fan blades or massive broken parts flung outward by the engine’s centrifugal force.
Aircraft tires	Kevlar^®^ brand aramid fiber	Enhanced toughness and thermal stability
Rotor blades	Nomex^®^ or Kevlar^®^ honeycomb core	Lighter, stiffer, and stronger than alloy cores.
Spacecraft	Kevlar^®^ fiber	Survive extreme forces and temperature fluctuations of space travel; Reinforce the inflatable landing cushions and ropes of the Mars Pathfinder.

### 2.2. Polymer Matrices for Aerospace AFRP

Composites are materials that are homogeneous on a macro scale, formed by a reinforcing phase dispersed in a relatively weaker material, known as the matrix [31]. The matrix can be metal, polymer, or ceramic, while the reinforcement takes the form of fibers, particles, or whiskers [32].

**Figure 2:**
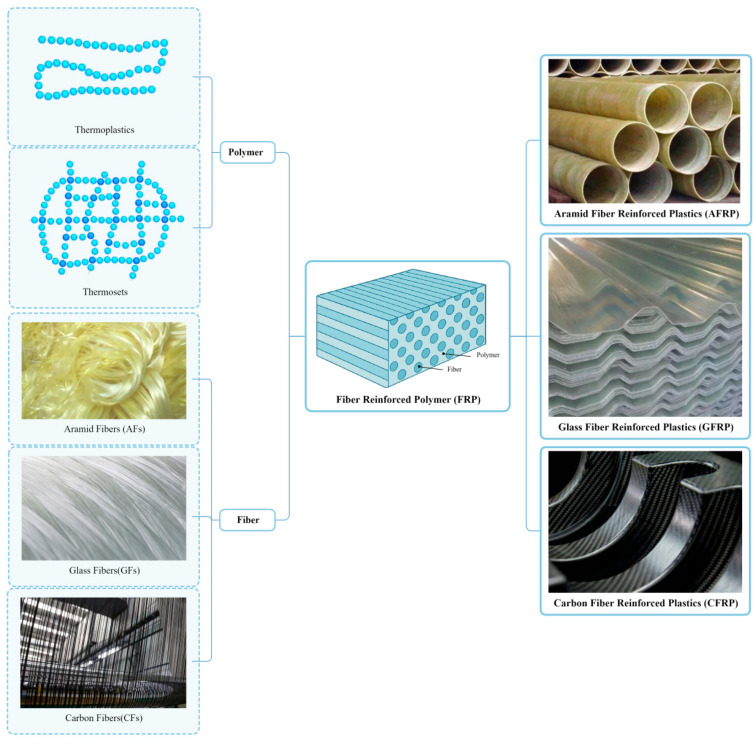
Structure and classification of FRP.

At present, fiber-reinforced polymer (FRP) is predominantly used in the aerospace field. FRP is an advanced material characterized by a high strength-to-weight ratio. According to the different fiber-reinforcing materials, FRP can be divided into aramid fiber-reinforced plastics (AFRPs), glass fiber-reinforced plastics (GFRPs), and carbon fiber-reinforced plastics (CFRPs), as shown in Figure 2.

The structure of FRP is illustrated in Figure 2, where the reinforcement (fibers) serves as the primary load-bearing elements, and the matrix encases the fibers, protecting them in the desired orientation. The matrix functions as a load-transfer medium between fibers and shields the structure from harsh environmental conditions, such as high temperatures and humidity [33].

In AFRP, the polymer matrix plays several key roles: it holds the aramid fibers together, maintains the fibers’ alignment, protects the fibers from environmental damage, and transfers loads between the fibers [34]. Therefore, the choice of matrix is crucial, as it directly affects the overall thermal performance, durability, manufacturing process, and cost of the composite. In the aerospace industry, both thermoset and thermoplastic polymers are widely used, with each material offering unique benefits and challenges.

Thermoset resins, which form rigid, cross-linked three-dimensional networks upon curing, have historically dominated the aerospace composites field. Epoxy resins are the most commonly used matrix material in aerospace-grade reinforced fiber-reinforced plastics (AFRPs) due to their balanced properties, including excellent adhesion to pre-treated aramid fibers, low shrinkage during curing, good mechanical properties over a wide temperature range, and outstanding chemical resistance [35,36]. However, the brittleness of epoxy resins limits the overall damage tolerance of the composites, and their lengthy and energy-consuming autoclave curing process not only poses significant manufacturing challenges but also significantly increases the cost of the final part [37]. For high-temperature applications, advanced thermoset resins, such as bis maleimides (BMIs) and polyimides (PIs) are commonly used, as they have higher glass transition temperatures but are often accompanied by increased brittleness and more complex processing requirements at elevated temperatures.

High-performance thermoplastics have emerged as an attractive alternative material due to their benefits in terms of increased productivity and enhanced sustainability. Polymers, such as polyether ether ketone (PEEK), and polyetherimide (PEI) offer superior toughness, inherent flame retardancy, and excellent damage tolerance compared to thermosets. More importantly, these materials enable faster manufacturing cycles through non-autoclave (OoA) processes, such as in situ reinforcement (strip placement) and stamping, and offer solutions to sustainability issues due to melt recyclability [38,39]. However, the main challenge with thermoplastic-reinforced fiber-reinforced composites (AFRPs) lies in the machining process. The high melt viscosity of these polymers makes it difficult to achieve complete non-porous impregnation of dense aramid fiber bundles, which remains a key area for process development [40]. Specifically, the increased viscosity limits the capillary-driven impregnation of aramid fiber bundles, resulting in insufficient fiber wetting and higher void formation [41]. This issue is worsened by the rapid crystallization of semi-crystalline thermoplastics (e.g., PEEK), which can lead to premature solidification before full impregnation occurs [41]. To address these problems, advanced processing techniques, such as in situ consolidation (ISC) and laser-assisted tape placement (LATP), have been examined, though they demand precise thermal control to avoid thermal degradation (e.g., PEEK’s processing window is 380–400 °C, near its degradation onset at approximately 440 °C) [41]. Furthermore, low-molecular-weight additives or reactive chain extenders have been suggested to lower melt viscosity, although they may adversely affect the final mechanical properties [42].

Compared to thermoset systems, thermoplastics demonstrate superior fracture toughness (e.g., CF/PEKK: GIC = 1.564 N/mm vs. CF/epoxy: 0.277 N/mm) and damage tolerance, but their processing complexity remains a barrier to large-scale adoption in the aerospace industry. The heterogeneous structure of AFRP further complicates machining, as the ductile thermoplastic matrix (strain-to-failure > 50%) sharply contrasts with the brittle aramid fibers, resulting in unique damage mechanisms such as matrix smearing and fiber pull-out [42].

### 2.3. The Critical Role of the Fiber–Matrix Interface

**Figure 3:**
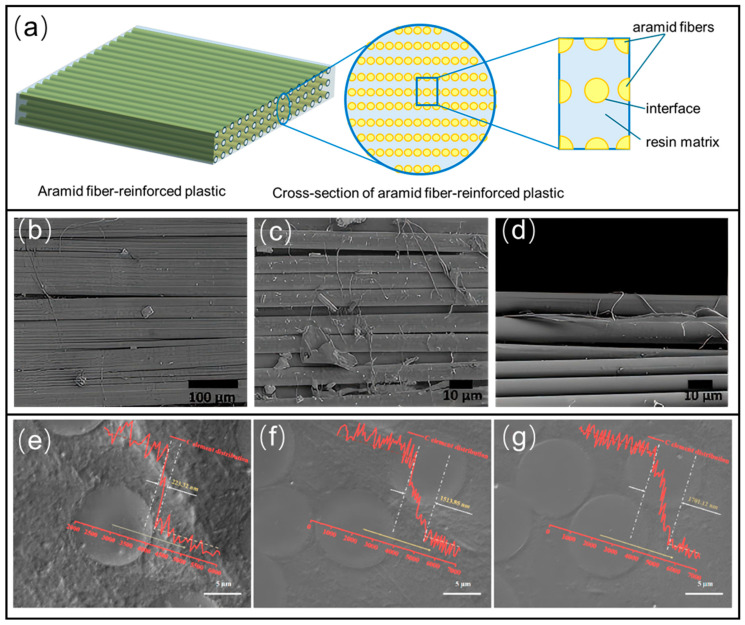
Cross-section of AFRP and fiber–matrix interface.(**a**) Conceptual schematic. Adapted from Ref. [43], MDPI, 2023. (**b**–**d**) SEM images of untreated aramid fabrics, after pullout test at yarn-crossing points. Reproduced from Ref. [44], Copyright John Wiley and Sons, 2019. (**e**–**g**) C element distribution in cross-section of composites: (**e**) AF/NR, (**f**) AF-PDES-2/NR, (**g**) AF-PDES-4/NR. Reproduced from Ref. [45], Copyright Elsevier, 2024.

As shown in Figure 3, the interface, the region where the fiber and matrix meet and interact, is not merely a boundary but a distinct and critical third phase within the composite. The performance of an AFRP is often won or lost at this interface.

Its primary role is to effectively transfer load from the lower-stiffness matrix to the high-strength aramid fibers. A robust interface is essential for realizing the full load-bearing potential of the fibers and preventing premature failure of the composite [46].

Specifically for AFRP, creating a strong and durable interface presents a unique scientific challenge. Unlike carbon fibers, which can form strong covalent bonds with epoxy matrices through surface functional groups, the chemically inert surface of aramid fibers results in an interface dominated by relatively weak physical interactions, such as van der Waals forces and mechanical interlocking [47,48]. This weak interfacial adhesion is directly responsible for many of the performance limitations of AFRP, including its poor transverse and compressive strength, as it fails to provide adequate lateral support to the fibers to resist microbuckling. Furthermore, a weak interface acts as a preferential pathway for moisture ingress, which can lead to interfacial debonding and accelerate the environmental degradation of the entire composite [49,50].

Therefore, engineering a strong, stable, and durable fiber–matrix interface is the most critical task in unlocking the full potential of AFRP. The strategies to achieve this, from nanoscale surface modification to the development of advanced matrix chemistry, form the core of the performance enhancement techniques discussed in the subsequent section.

## 3. Performance Enhancement of AFRP

In Section 2, a fundamental link between the molecular structure of aramid fibers and the performance of AFRP has been established. This section will critically evaluate the key strategies being developed to overcome the inherent limitations of the material. The following section will delve into core research areas that aim to bridge the gap between the theoretical potential of AFRP and its practical applications. How targeted interventions can be implemented at the fiber–matrix interface, within the material, and through the integration of novel functionalities will be explored to pave the way for the development of a new generation of high-performance aramid composites.

### 3.1. Interfacial Engineering: Strategies and Mechanisms

According to Section 2.3, aramid fibers, due to their chemical inertness and physical smoothness, result in a weak interface between the fibers and the matrix, which is the primary reason for the poor performance of the composites, especially their low compressive and transverse strengths [51]. Therefore, constructing a strong and durable interface is a key strategy to unlock the full potential of AFRP. Research in this field is mainly divided into physical and chemical modification techniques, each of which aims to enhance adhesion through different mechanisms. A comparison of the various surface modification techniques is shown in Table 4.

#### 3.1.1. Physical Surface Modification

Physical methods alter the fiber’s surface topography and surface energy without changing its bulk chemical composition. These techniques are often favored for their relative simplicity and solvent-free nature.

Plasma surface modification is a prominent dry-processing technique that utilizes high-energy ionized gas to bombard the fiber surface, creating active radical sites, introducing polar functional groups, and increasing surface roughness through nanoscale etching [52]. This enhances both mechanical interlocking and chemical reactivity with the resin matrix. For example, Xu et al. [52] demonstrated that an optimized atmospheric air plasma treatment (10 min at 400 W) on aramid fibers increased the surface’s active group content by 82.4% and improved the interlaminar shear strength (ILSS) of the resulting epoxy composite by a remarkable 45.5%. Ehsan Shakerinasab et al. [53] used argon as the primary working gas and toluene, acetonitrile, tetraethyl orthosilicate (TEOS), and hexamethyldisiloxane (HMDSO) as liquid precursors for plasma coating. Their experimental results indicated that atmospheric plasma coating with acetonitrile precursors can increase the bond strength between aramid fibers and polymeric matrices for composite applications.

However, plasma treatment is not without its challenges; the same study noted an 8.6% loss in the fiber’s tensile strength, highlighting the critical need for precise process control to prevent surface damage [52]. Furthermore, ensuring uniform treatment depth and long-term durability of the modified surface, especially on large or complex 3D components, remains a significant challenge for industrial-scale aerospace applications [52,57]. Batch-to-batch uniformity quickly declines when treating aerospace-scale preforms because the outer filaments in a tow receive higher ion flux than inner filaments, leading to uneven oxidation and potential hot spots that weaken fibers locally [58]. Continuous roll-to-roll DBD units need nitrogen purging or vacuum chambers to prevent arcing at high web speeds, which increases both capital costs and cycle time beyond the 30–60 s dwell typical of autoclave prepreg lines [59]. As a result, plasma is currently limited to niche aerospace tapes or research coupons rather than full-scale fuselage skins.

#### 3.1.2. Chemical Surface Modification

Chemical methods involve altering the fiber’s surface chemistry to introduce functional groups that can form strong covalent or hydrogen bonds with the matrix. Among the most effective strategies are surface etching, silane grafting, and hybrid nanoscale sizing, each offering distinct mechanisms for energy dissipation and toughness enhancement.

Chemical Etching and Grafting: chemical etching uses reagents to remove material from the fiber surface, increasing roughness selectively. Lin et al. [60] demonstrated that sequential treatment with CaCl_2_ and NaOH etched aramid fibers, increasing surface roughness and enabling subsequent silane coupling (KH570) grafting, which improved the tensile strength of NR/BR composites by 31.9% and abrasion resistance by 16.2%. This mechanism is attributed to the synergistic effect of mechanical interlocking and chemical bonding formed between the silanol groups of KH570 and the hydrolyzed fiber surface [60]. While effective, this approach often involves harsh chemicals, posing environmental and safety concerns for industrial adoption [54]. Silane grafting via γ-ray irradiation offers a more controlled and effective approach. Jia et al. utilized γ-ray irradiation for grafting amino silane (APS) onto aramid fibers, resulting in a 51.03% increase in interfacial shear strength (from 36.33 MPa to 54.87 MPa) in an epoxy composite, crucially without compromising the fiber’s tensile strength [55]. The grafted amino groups form covalent bonds with epoxy matrices, while irradiation-induced surface roughening enhances mechanical interlocking. The energy dissipation mechanism involves crack bridging by the grafted layer and stress redistribution through covalent bonding, delaying interfacial debonding under cyclic loading [55]. The industrial translation of chemical surface modification is limited by quantifiable constraints directly stemming from published datasets. Palola et al. [59] reports that multi-step acid/alkali etching uses 3–5 L of concentrated NaOH (5 M) and 2 L of CaCl_2_ solution per kilogram of aramid fiber; neutralization to pH 7–8 produces approximately 8 L of saline effluent containing residual Cr(VI) and phenolic by-products, increasing disposal costs to between 0.8 and 1.2 USD per kilogram of treated fiber—significantly higher than the typical 0.3 USD per kilogram in aerospace composite plants. Du et al. [58] adds that γ-ray grafting at the established 600 kGy dose requires a Co-60 source with an activity of 2.5 × 10^16^ Bq to support a 1 ton per hour throughput. The necessary 2-m concrete shielding vault costs around USD 3 million and is regulated under IAEA Category II rules, which adds 6–9 months for licensing.

Bio-Inspired and Nanoscale Coatings: drawing inspiration from nature, researchers have developed innovative coating methods. A prominent example is the use of mussel-inspired polydopamine (PDA) coatings. Zhang et al. deposited a thin layer of tannic acid (TA) and polyethyleneimine (PEI), which mimics the adhesive proteins of mussels, onto the fiber surface. This was followed by coating with aramid nanofibers (ANFs) to create a hierarchical nanostructure, which enhanced interfacial adhesion in a rubber composite by up to 43.1% [55]. This bio-inspired approach is attractive due to its mild, aqueous-based processing conditions.

Coupling Agents and Hybrid Sizing: Coupling agents, such as silanes, serve as molecular bridges between the fiber and the matrix. A more advanced strategy involves creating hybrid sizing agents. Sharma et al. [61] developed a 3D graphene-carbon nanotube (GCNT) hybrid sizing for aramid/polycarbonate composites, achieving 32% higher tensile strength (367 MPa vs. 278 MPa baseline) and 188% increased storage modulus (7.8 GPa vs. 2.7 GPa). The GCNT network bridges adjacent aramid fibers, dissipating energy through crack deflection and nanotube pull-out, while graphene sheets encapsulate fibers to enhance load transfer. This multi-scale architecture delays crack propagation by combining covalent bonding (via silane) and mechanical interlocking (via GCNT), significantly improving toughness [61]. Zhou et al. developed a sizing composed of a soluble polyimide (PI) compatibilizer, rigid carboxyl-functionalized carbon nanotubes (CNT-COOH), and flexible aramid nanofibers (ANF) [56]. This multi-scale structure significantly improved the interfacial bonding with a PEEK matrix, resulting in increases of 132.6% in flexural strength and 99% in flexural modulus [56]. This demonstrates the immense potential of multi-scale interfacial design. However, the high cost of nanomaterials, such as CNTs, and the challenge of achieving their uniform dispersion at an industrial scale currently represent significant barriers to the widespread aerospace application of such advanced hybrid systems.

Multi-wall CNT prices are 150–300 USD kg^−1^, and achieving a 0.5 wt% CNT loading in 10,000 kg of epoxy necessitates 50 kg of CNTs (USD 7500–15,000)—compared with ~USD 200 for conventional silane sizing—while high-shear dispersion at 3000 rpm for 60 min raises batch viscosity to 8000 mPa·s, exceeding the 5000 mPa·s limit of standard aerospace prepreg impregnation lines [62,63]. Consequently, these data-driven constraints confine nanoscale hybrid coatings to laboratory coupons (<1 kg) rather than flight hardware (>100 kg).

### 3.2. Overcoming Mechanical Deficiencies

While a strong interface is crucial, addressing the specific mechanical weaknesses of AFRP—particularly their low compressive strength and limited fatigue life under cyclic loading—requires targeted strategies. AFs have a low shear modulus, lack lateral properties, and exhibit low compressive strength because their rod-like molecular chains are not parallel. There are many hydrogen bonds between polar amide groups on adjacent chains that are perpendicular to the fiber axis. These hydrogen bonds are weaker than the covalent bonds in the fiber direction. The lateral strength of the fiber is about 20% of its longitudinal strength. Early research by Piggot and Harris compared the compressive strengths of carbon fiber, glass fiber, and Kevlar-49-reinforced polyester resin. At a volume fraction of 30%, the compressive modulus and strength of Kevlar fiber composites are significantly lower than those of their tensile counterparts [64]. This failure mode occurs when a weak interface fails to adequately restrain [64]. Therefore, enhancing compressive performance is intrinsically linked to both improving the interface and modifying the material’s micro- and macro-structure. Quantitative evidence now shows that fatigue life under cyclic loading must be concurrently optimized for aerospace structural use.

At the microscopic level, enhancing intermolecular interactions between polymer chains can greatly improve stress transfer efficiency and increase the compressive strength of aramid materials [65]. Introducing covalent cross-linking is a common method used for this purpose [66]. Deng et al. [23] created branched multi-hydrogen bonding sites within the fibers using nano-silica, which simultaneously raised the axial and transverse compressive strengths by 68.8% and 26.8%, respectively. This work emphasizes that strengthening the inherent lateral cohesion between polymer chains is a key mechanism for resisting micro-buckling, and the APTMS-grafted nano-silica approach used by Dharmavarapu and Reddy [67] delivers the same mechanism while also suppressing fatigue-crack initiation via nano-particle-induced crack pinning and load redistribution, as confirmed by the absence of interfacial cracking in SEM fractographs. In this study, Kevlar-49/epoxy laminates with 40 vol.% aramid fiber and 1 vol.% APTMS-silane-modified nano-silica (named N_3_) showed a tensile fatigue life of 16,391 cycles at 5 Hz, R = 0.1, and 23 °C, nearly doubling the 9001 cycles seen in the fiber-only control (N_1_) and exceeding the 629 cycles of neat epoxy (N0) [67]. The same 1 vol.% nano-silica formulation also achieved tensile and flexural strengths of 102 MPa and 135 MPa, respectively, while THE Izod impact energy reached 6 J, demonstrating that a single low-dose nano-additive can concurrently enhance static strength, impact resistance, and cyclic durability [67].

At the macroscopic level, architectural modifications offer a powerful route to performance enhancement. Three-dimensional (3D) weaving, which introduces through-thickness reinforcement, is particularly effective. Li et al. [68] demonstrated that combining surface modification with a 3D woven architecture increased the compressive modulus by 124% and the shear strength by 55.7% compared to unmodified 2D composites [68]. Kang et al. [69] chose AF and PTFE, which have good mechanical strength and heat resistance, to develop a 3D AF/PTFE composite. Compared to the traditional 2D composite, its mechanical properties were significantly improved, and the wear rate was reduced by 44%. The 3D weaving directly addresses the weakness of traditional 2D laminates—their susceptibility to delamination—by mechanically interlocking the layers, thereby providing greater support against buckling and significantly improving damage tolerance. Despite these advantages, 3D architectures introduce notable drawbacks, including elevated void content and stress concentrations that can compromise structural integrity in aerospace applications. For instance, the complex weave geometries and through-thickness fibers in 3D structures hinder resin impregnation, leading to higher void formation (typically 3.5–5.5% by volume, compared to 0.8–1.2% in 2D laminates), which arises from air entrapment and poor matrix flow, exacerbating risks by over 40% relative to 2D counterparts [70,71]. These voids act as initiation sites for damage, reducing compression strength by 5–10% per 1% void increase and weakening interfacial shear strength by 20–30% due to material incompatibility between hydrophilic aramid fibers and hydrophobic matrices [70]. Additionally, stress concentrations at weave junctions and z-bindings promote fatigue failure under cyclic loading, with mechanisms, such as matrix microcracking and debonding, resulting in 50–60% faster delamination growth and a 25–40% reduction in flexural fatigue life compared to 2D laminates [72]. Mitigation strategies, such as optimizing resin viscosity (300–500 cP) and infusion pressure (>4 bar), or employing topology optimization to reduce stress intensity by 35%, are essential to balance these trade-offs [70,73]. At the same time, the complexity and cost of 3D weaving processes are higher than those for 2D laminates, representing a trade-off that the required performance gain in a specific application must justify.

Taken together, the dual strategy of nano-silica toughening and 3D weaving not only enhances compressive and shear properties but also ensures a fatigue life exceeding 1.6 × 10^4^ cycles, meeting a critical design requirement for aerospace structures exposed to variable-frequency loading.

### 3.3. Bolstering Environmental Durability

For AFRP to be reliably used in long-life aerospace structures, its performance must be stable across a wide range of harsh environmental conditions [74]. Key efforts focus on mitigating degradation from thermal exposure, moisture, and ultraviolet (UV) radiation.

#### 3.3.1. Thermal Degradation

The thermal degradation of AFs primarily occurs through the cleavage of specific chemical bonds. Studies have shown that the decomposition of aramid fibers typically begins with the rupture of hydrogen bonds, followed by the cleavage of amide bonds, such as the C=O and C-N bonds [75]. The initial stages of decomposition are characterized by the loss of hydrogen bonds and minor structural changes, whereas significant weight loss occurs at temperatures above 500 °C [75]. While the aramid fiber itself is thermally stable at temperatures exceeding 400 °C, the composite’s service temperature is often limited by the polymer matrix.

To enhance the thermal durability of AFRP, one strategy involves stabilizing the interface between the resin and the fiber. Al-Quraishi et al. used polymer-derived ceramics (PDCs) as a coating for fibers. During heating, the PDCs form a ceramic network at the interface, yielding an interfacial shear strength (IFSS) at 100 °C comparable to that of untreated fibers at room temperature [76]. This demonstrates that a thermally stable interface is critical for retaining mechanical properties at elevated temperatures.

Additionally, the use of advanced polymer matrices with higher glass transition temperatures (Tg) can improve the thermal stability of AFRP. High-performance polymers, such as polyimides and bismaleimides, offer better thermal resistance compared to traditional epoxy resins. These matrices can maintain their mechanical properties at higher temperatures, thereby extending the operational temperature range of AFRP [77]. This demonstrates that a thermally stable interface is critical for retaining mechanical properties at elevated temperatures.

#### 3.3.2. Moisture Absorption

AFs are hydrophilic, and moisture absorption can plasticize the matrix, lower its glass transition temperature (Tg), and degrade the fiber–matrix interface [78]. To mitigate the adverse effects of moisture absorption on AFRP, several strategies have been proposed. Recent approaches to combat this involve creating a hydrophobic barrier at the nanoscale. Protyai et al. demonstrated that incorporating graphene, SiC, or Al_2_O_3_ nanoparticles into the epoxy matrix can significantly reduce water uptake and enhance the thermal stability of Kevlar composites [79]. While effective, achieving uniform dispersion of nanoparticles without agglomeration, which can create new defect sites, is a persistent manufacturing challenge.

Furthermore, modifying the epoxy resin matrix with flame-retardant additives can also enhance the moisture resistance of AFRP. Phosphorus-containing compounds, such as the multifunctional flame-retardant modifier EAD, have been shown to improve interfacial bonding and reduce moisture absorption by forming a protective char layer during combustion [80]. This modification not only enhances the flame retardancy of the composite but also improves its mechanical properties and ballistic performance [80].

#### 3.3.3. UV Radiation

AFs, with numerous amide groups and highly aligned crystalline molecular chains, degrade after short ultraviolet (UV) exposure [81]. Studies indicate that these changes are largely driven by photo-oxidative reactions in air, which mainly affect the amorphous and defect-rich zones, whereas the bulk crystalline phase remains relatively stable with only minor local adjustments. The loss of mechanical integrity is mainly associated with surface microstructural damage and a reduction in the order along the fiber axis, rather than wholesale disruption of the crystalline lattice [82]. These reactions result in a reduction of tensile strength by about 20–30% after 168 h of UVB irradiation (40 Wm^−2^) for untreated AFs, along with surface roughening, discoloration, and a 50% decrease in modulus [83]. Recent studies have shown that 168 h of UV-B irradiation (40 W m^−2^, 280–315 nm) decreased the tensile strength of untreated Kevlar^®^ by about 20% [83]. However, new surface-engineering approaches have significantly improved the durability of AF-based composites.

Applying UV absorbers or applying UV-resistant coatings or incorporating UV absorbers can enhance UV resistance. In AFRP composites, adding UV absorbers can protect materials by converting UV energy into harmless heat or other forms of energy. An approach involves synthesizing TiO_2_ or ZnO nanoparticles using Sc-CO_2_ and coating fibers with them, which not only enhances UV resistance but also improves fiber-coating adhesion and durability [84]. Zhang et al. [85] optimized the ZnO deposition by coordinating Zn^2+^ directly with AF in a Sc-CO_2_ drying step, forming C–O–Zn bonds that raise grafting density to 34.23 % and elevate IFSS by 68.2 % while preserving 93.1 % tensile strength after 216 h UV-B irradiation. The same ZnO-bonded fibers also exhibited concurrent improvements in tensile strength (+13.7 %), modulus (+8.7 %), elongation at break (+13.4 %), and fracture energy (+15.7 %), indicating that the coordination chemistry approach mitigates UV degradation without compromising intrinsic mechanical or thermal stability. 

Amorphous TiO_2_ nanocoatings (2.38 wt%, 15 MPa Sc-CO_2_), produced via supercritical CO_2_ (Sc-CO_2_) infiltration, reduced tensile strength loss to about 14% after 168 h of UV exposure, outperforming untreated fibers [83]. The protective effect stems from TiO_2_’s UV-absorbing ability (λ < 400 nm) and its capacity to scatter incident radiation, confirmed by UV-Vis spectroscopy showing 30% lower absorbance at 320 nm for TiO_2_-modified fibers [83]. Similarly, dual-layer Al_2_O_3_–TiO_2_ coatings (70–180 nm) deposited through modified atomic layer deposition (ALD) display excellent UV resistance: tensile strength decreased by only 0.85% after 90 min of exposure to 4260 W/m^2^ UV light at temperatures above 200 °C, simulating 17,500 days of natural sunlight [16]. The inner Al_2_O_3_ layer (37.4 nm) functions as a radical scavenger and diffusion barrier, preventing UV-generated reactive oxygen species (ROS) from reaching the fiber core. Meanwhile, the outer TiO_2_ layer (58.7 nm) absorbs UV via bandgap excitation (Eg ≈ 3.2 eV), as indicated by FTIR showing minimal formation of degradation products, such as carbonyl (C=O, 1715 cm^−1^) or hydroxyl (–OH, 3400 cm^−1^), after irradiation [16].An alternative approach involves dispersing UV-stable nano-fillers evenly throughout the matrix. Glass-fiber/epoxy composites with 0.5 wt% MWCNTs showed a 90% decrease in micro-cracking after 2160 h of UV-A exposure [86].

These strategies balance the trade-off between UV resistance and photocatalytic activity: amorphous TiO_2_ coatings produce less ROS compared to crystalline anatase, while Al_2_O_3_ underlayers reduce electron-hole recombination in TiO_2_. Overall, surface-engineered AFs via Sc-CO_2_ or ALD provide scalable, damage-free ways to extend AFRP service life in extreme UV environments, maintaining over 90% of mechanical properties under accelerated aging [16,83]. In summary, surface functionalization with ZnO/TiO_2_ nanostructures and matrix-level addition of UV-stable fillers or encapsulants provide effective methods to mitigate UV-induced degradation in AFRP, ensuring long-term durability without compromising the inherent properties of AFs.

### 3.4. Development of Functional AFRP

To enhance their value proposition in an increasingly competitive materials landscape, the latest research thrust is to evolve AFRP from purely structural materials into multifunctional systems that can sense, adapt, and self-repair [87,88,89].

#### 3.4.1. Shape Memory AFRP

**Figure 4:**
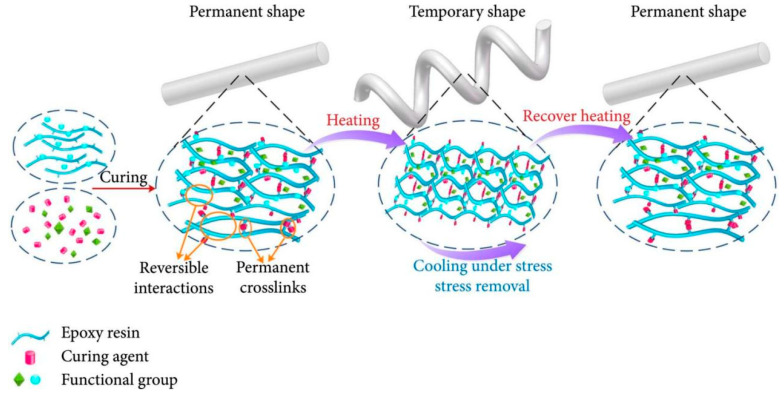
Schematic diagram of the shape memory mechanism of epoxy resin. Reproduced from Ref. [94], AAAS, 2022.

Shape memory polymers (SMPs), novel innovative materials that can respond to external stimuli and revert to their original shape, can be activated not only by thermal and magnetic means, like shape memory alloys (SMAs), but also by electrical, optical, and humidity triggers and even specific chemical stimuli, such as pH changes [90]. SMPs offer several potential technical advantages over SMAs and shape memory ceramics. These include excellent shape recoverability (with recoverable strains as high as 400%), low density, ease of processing, and customizable properties (such as transition temperature, stiffness, biodegradability, and ease of function grading), as well as programmable and controllable recovery behavior. Most importantly, SMPs are cost-effective [91]. Nevertheless, SMPs have drawbacks, such as low deformation stiffness and recovery stress. To address these issues, shape memory polymer composites (SMPCs) have emerged, featuring enhanced strength and stiffness, along with unique properties determined by the type of fillers used [92]. The applications of SMPCs in the aerospace field are extensive, including components, such as SMPC hinges, reflective antennas, and morphing wings, as well as structures, like solar cell arrays and deployable panels [93]. The shape memory mechanism is illustrated in Figure 4.

Epoxy resins (EP), with advantages, such as high stiffness, good adhesion to various substrates, and good thermal and chemical resistance, are widely used in the aviation and aerospace industries. Shape memory epoxy resins (SMEP) are no different and are also widely used in these fields for the same reasons [95]. The shape memory effect in most SMEPs is based on two structural characteristics: cross-linking, which determines the permanent shape, and a transition temperature (T*_trans_*), which fixes the temporary shape [91]. Zhang et al. [87] prepared shape memory epoxy-based AFRP and measured a tensile modulus of 73.58 GPa for it. The AFRP also had a shape fixity rate as high as 98.42%, a shape recovery rate of 79.84%, and a shape recovery force of 6.56 N. These characteristics enable self-deploying space structures. Such materials are envisioned for self-deploying aerospace structures, such as satellite antennas or solar arrays, which can be compactly stowed for launch and then autonomously deployed in orbit, eliminating the need for heavy and complex mechanical systems [93].

#### 3.4.2. Self-Healing AFRP

The service life of AFRP used in aerospace is often compromised by microcracking and delamination, especially under cyclic loading and environmental erosion. Self-healing AFRP, leveraging dynamic chemical bonds, offers a promising solution to enhance durability and reduce maintenance costs.

The concept of self-healing in AFRP primarily revolves around two approaches: extrinsic and intrinsic self-healing systems [96].

Extrinsic systems involve incorporating healing agents, such as microcapsules or vascular networks, into the polymer matrix. These agents release healing substances upon damage, which then polymerize to repair the matrix [96]. Upon crack initiation, the reservoirs rupture or open, releasing liquid healing agents that then polymerize and re-bridge the damaged interfaces. Epoxy–solvent microcapsules, for example, have achieved a record healing efficiency of 3500% in tapered double-cantilever beam (TDCB) epoxy specimens, demonstrating the effectiveness of this strategy [97]. Embedding 20 wt% DCPD-filled urea–formaldehyde microcapsules along with 2.5 wt% wax-protected Grubbs’ catalyst increased the virgin Mode-I fracture toughness (KIC) of an epoxy matrix by approximately 25% but reduced the flexural modulus by 8% and the interlaminar shear strength (ILSS) by 12% compared to the neat resin. Cyclic loading studies further showed a progressive performance decline: after five successive crack-opening/closing cycles, healing efficiency dropped from 90% to 60%, with an additional 18% decrease in KIC attributed to capsule depletion and residual voids [98]. Consistent results were reported by Takayama et al. [99], who used short carbon nanofibers (CNF) as a substitute for microcapsules: adding 5 wt% CNF to injection-molded aramid/PP composites reduced ILSS by 9% and weld strength by 7%, with the loss attributed to resin-rich defect bands surrounding the inclusions.

However, including foreign phases naturally causes mechanical penalties. A self-healing composite with 14 wt.% microcapsules can achieve the best results [100]. 

Microencapsulated healing agents show impressive mechanical performance and regenerative capacity, but they are limited to autonomic repair of a single damage event in one location. Self-healing occurs when cracks rupture the embedded capsules; once a localized area runs out of healing agent, further repair is impossible. Re-mendable polymers can undergo multiple healing cycles, but they require external intervention such as heat treatment and pressure [101]. Microvascular networks offer an alternative route for repeated healing [101]. Wang et al.’s work on GFRP laminates shows that, without intervention for light repair, the average maximum failure load of the self-healing structure after embedding the microvascular network can reach 94.06% of its original value [102]. Due to the similar structure of AFRP laminates, a comparable mechanical penalty is expected. Overall, the data show that extrinsic self-healing strategies greatly improve damage tolerance, but their integration into AFRP must be carefully optimized to reduce losses in stiffness and strength. Intrinsic self-healing, on the other hand, relies on the inherent properties of the polymer matrix, such as reversible covalent bonds or supramolecular interactions, which can reform upon damage [103]. Dynamic covalent bonds, such as those based on Diels–Alder (DA) chemistry, have shown significant potential in self-healing AFRP. The DA reaction involves the formation of thermoreversible bonds between electron-rich dienes (e.g., furans) and electron-poor dienophiles (e.g., maleimides). When heated above 120 °C, these bonds break, allowing the polymer chains to reorganize and reconnect upon cooling, thereby facilitating repair [104]. A novel advancement comes from chemically recyclable polyurea plastics (PUHA), which utilize hemiaminal groups for reversible crosslinking [105]. These plastics not only exhibit excellent mechanical properties (e.g., PUHABAPP with a Young’s modulus of ~1128.5 MPa and PUHABBA with a toughness of ~104.3 MJ/m^3^) but can also be depolymerized into linear polyurea under acidic conditions. When applied as a matrix for AFRP, the composites achieve high tensile strength (~215.6 MPa for PUHABBA/AF and ~253.2 MPa for PUHABAPP/AF) and demonstrate excellent puncture resistance. More importantly, the intact aramid fibers can be non-destructively recycled from the composites through acid immersion, addressing the critical challenge of material recyclability [105].

In conclusion, self-healing AFRP represents a significant step forward in addressing the challenges of damage mitigation in aerospace structures. While progress has been made in developing intrinsic and extrinsic self-healing systems, further research is needed to optimize mechanical performance, enhance healing efficiency, and ensure manufacturability and reliability for aerospace applications.

#### 3.4.3. Sensing AFRP

The integration of sensing capabilities into AFRP has been a key focus for enhancing structural health monitoring (SHM) in aerospace applications [106]. Recent advancements have demonstrated the potential of AFRP to provide not only mechanical strength but also enable real-time damage detection and monitoring through embedded sensors [107]. Among the various sensing methodologies, the use of aramid fibers themselves as strain sensors has gained significant attention due to their piezoresistive properties.

By utilizing the Raman response of aramid fibers, researchers have developed non-destructive methods to evaluate interface integrity and overall stress distribution in unidirectional Kevlar-29/epoxy composites. This approach has proven effective in examining damage development in composites with various length-scale discontinuities, such as fiber breaks and voids [107]. Micro-Raman spectroscopy on single Kevlar-29 filaments embedded in epoxy microdroplets (D = 12 µm) shows that applying a 0.77% strain causes a −3.1 cm^−1^ shift in the 1611 cm^−1^ Raman peak, indicating a local fiber stress of 425 MPa [108]. The same technique quantifies how interface geometry influences stress transfer: the characteristic transfer length decreases from 160 µm to 90 µm, and the peak interfacial shear stress increases from 18 MPa to 41 MPa as the droplet edge angle rises from 21° to 73° [108]. These calibrated parameters can be directly applied to notched Kevlar-49/epoxy laminates, where Raman mapping with 20 µm spatial resolution tracks stress concentrations and validates shear-lag predictions within ±5% [109]. As a result, the Raman-enabled AFRP functions as a spatially resolved, non-destructive strain sensor capable of detecting damage initiation and progression at the ply level without additional embedded hardware. The ability to monitor these parameters in real time is vital for ensuring the safety and reliability of aerospace structures.

In addition to strain sensing, aramid fibers have been explored for their potential in energy harvesting applications. The piezoelectric effect of certain innovative materials enables the conversion of mechanical energy into electrical energy, which can be harnessed to power sensors or other electronic components embedded within the composite structure [110]. This self-powered sensing capability further enhances the multifunctionality of AFRP, reducing the need for external power sources and making it an attractive option for aerospace applications where weight and power consumption are critical concerns.

The development of self-sensing hybrid FRP composites has also been investigated, with carbon fiber sensors embedded within the composite material enabling real-time damage detection. Long short-term memory (LSTM) neural networks have been implemented to predict remaining fatigue life using only resistance data, eliminating the need for stress or strain inputs. This approach has demonstrated strong correlations between resistance and cycles to failure, offering a novel method for predicting fatigue life in AFRP composites. The carbon fiber sensor tows have demonstrated the ability to provide damage early warnings through a sharp increase in resistance before failure, highlighting the potential for a simple, low-cost SHM system that can significantly extend the service life of composite structures [111].

Furthermore, the combination of laser-induced graphene (LIG) creates a conductive network that enables real-time monitoring of damage initiation and propagation [112]. By integrating laser-induced graphene (LIG) onto Kevlar KM2+ fabric using a CO_2_ infrared laser before epoxy infusion, a lightweight, co-cured conductive network is formed within the composite. Coupons 11–14 mm wide and 85–110 mm long act as intrinsic strain sensors: single-sided LIG produces baseline resistances of 60–860 Ω, while double-sided LIG stabilizes at 60–120 Ω. Under monotonic tension, the laminates provide an average gauge factor of 0.81 up to 3% strain, maintaining this sensitivity without drift across five load–unload cycles [88]. The smart composite continuously monitors interlaminar and tensile damage through changes in resistance, enabling self-diagnosis without adding extra mass or external sensors.

In summary, the integration of sensing capabilities into AFRP has opened up new avenues for SHM in aerospace applications. From strain sensing and energy harvesting to fatigue life prediction and damage detection, these multifunctional composites are pushing the boundaries of what is possible in terms of structural performance and reliability. As research continues to evolve, the potential for AFRP to revolutionize the aerospace industry by providing safer, more durable, and intelligent structures becomes increasingly evident.

### 3.5. Computational Modeling for Performance Prediction and Design Optimization

Alongside experimental studies, computational modeling has become a vital tool in analyzing AFRP [113]. Acting as a crucial link between microscopic mechanisms and overall performance, simulations enable faster development of new materials through virtual testing. They also provide theoretical insights for designing complex structures, thereby reducing the reliance on costly and time-consuming physical experiments.

#### 3.5.1. Multi-Scale Modeling Approaches

Considering the inherently complex and hierarchical structure of AFRP, multi-scale modeling approaches are essential to accurately capture their key behaviors across different length scales.

**Molecular Dynamics (MD) Simulations:**

At the atomic and molecular levels, MD simulations investigate the fundamental interactions at the fiber–matrix interface. By constructing atomistic models with aramid molecular chains and resin molecules, MD can evaluate interfacial adhesion energies, shear strengths, and analyze how environmental factors, such as moisture or temperature fluctuations, impact interfacial bonding [114]. These simulations provide a theoretical foundation for interfacial engineering strategies—such as those described in Section 3.1—by predicting how various functional groups can enhance interface adhesion.Recently, Zhang et al. [115] performed all-atom MD and correlation analyses in order to understand the microstructural evolution and property improvement of GO-PPDA/AF, and prepared AFs with high tensile strength (6.75 GPa) and high compressive strength (676.8 MPa).

**Micromechanics Models:**

At the micrometer scale, representative volume element (RVE)-based micromechanics models are frequently employed. By accurately replicating the geometric arrangement of fibers within an RVE, these models predict the effective mechanical properties, including elastic modulus, Poisson’s ratio, and strength, of basic composite units, like unidirectional laminates and woven fabrics. Importantly, RVE models also enable simulation of damage initiation processes, especially the critical compressive failure mode in AFRP: fiber micro-buckling. This allows for detailed analysis of relationships between micro-buckling, initial fiber imperfections, matrix characteristics, and interfacial strength [116].

**Macro-scale Finite Element Analysis (FEA):**

At both component and structural levels, macro-scale FEA forms the basis of engineering design. Using homogenized material properties derived from micromechanics models, FEA simulates how entire components respond mechanically under complex loading conditions—including stress distribution, buckling, vibration modes, and damage evolution. Incorporating progressive damage models, FEA can forecast the entire failure process of composite structures, from initial damage to ultimate rupture [117].

#### 3.5.2. Simulation and Prediction of Key Behaviors

Multi-scale computational simulations have been successfully used to predict various critical behaviors in AFRP.

**Mechanical Performance Prediction:**

Computational models effectively forecast AFRP behavior under tensile, compressive, shear, and impact loads. Notably, simulating complex compressive failure modes—such as fiber micro-buckling and kinking—and post-impact compressive (CAI) performance clarifies how internal damage, like delamination, influences residual strength, offering vital input for damage-tolerant design [118].

**Environmental Aging Simulation:**

By solving Fick’s diffusion equations, models can simulate moisture ingress and its spatial distribution within composites. When combined with mechanical models, they help predict how stiffness and strength degrade under combined hygrothermal aging, supporting assessments of long-term durability in demanding service conditions [119].

**Processing Simulation:**

Computational models also simulate manufacturing processes, complementing insights from Section 4. For example, modeling resin flow and chemical reactions during curing helps predict residual stresses caused by thermal mismatch and chemical shrinkage, which is crucial for controlling the final shape and dimensional accuracy of composite parts [120].

Despite these advancements, several challenges remain in computational modeling. Key issues include: (1) effectively integrating different length scale models—“handshaking”—to accurately transfer information; (2) developing and validating constitutive models that reliably capture complex damage processes; and (3) the high computational cost associated with high-fidelity simulations.

## 4. Advanced Manufacturing Technologies for Aerospace AFRP

The transition of AFRP from specific applications to a wide range of uses in aerospace structures is closely linked to advances in manufacturing technology. Despite its flexibility, the traditional hand layup process is inefficient, costly, and of inconsistent quality and cannot meet the modern aerospace industry’s requirements for production speed and reliability. This section will delve into advanced manufacturing technologies that enable cost-effective, consistent quality, and scalable production of aerospace-grade AFRP. We will focus on three core areas: automated layup techniques, designed to address both speed and accuracy; curing and reinforcement innovations, critical to achieving optimum material performance; and the emerging field of additive manufacturing, which offers unprecedented design freedom.

### 4.1. Automated Layup Technologies (AFP, ATL, AFW): Principles, Advancements, and Challenges

The conventional hand layup molding process forms a preform by manually laying fabrics and resin layer-by-layer on the mold surface. It has low equipment requirements but high labor costs and low efficiency. Moreover, the products often have many voids and wrinkles, leading to performance below the design objectives. Consequently, its popularity in the aerospace industry is decreasing.

To enhance production efficiency and reduce defects in composite structures, computer and machine-assisted automatic layup technology (ALT) has been introduced to accelerate the layup process. ALT includes automatic fiber winding (AFW), automatic tape laying (ATL), and automatic fiber placement (AFP). Alexander Eremin et al. [121] examined how different layup schemes affect the mechanical properties of AFRP samples. The flexibility, high deformation potential, and woven fabric structure of aramid fibers lead to deformation localization in fiber-tow regions at low strain levels due to the combined effects of these factors. The load is well-distributed across the sample area by the flexible aramid fibers. Aramid composites easily reach the nonlinear stage, relaxing external loads due to significant local inelastic deformation. Table 5 summarizes composite preforming methods and their aerospace applications.

#### 4.1.1. Automatic Fiber Winding (AFW)

**Figure 5:**
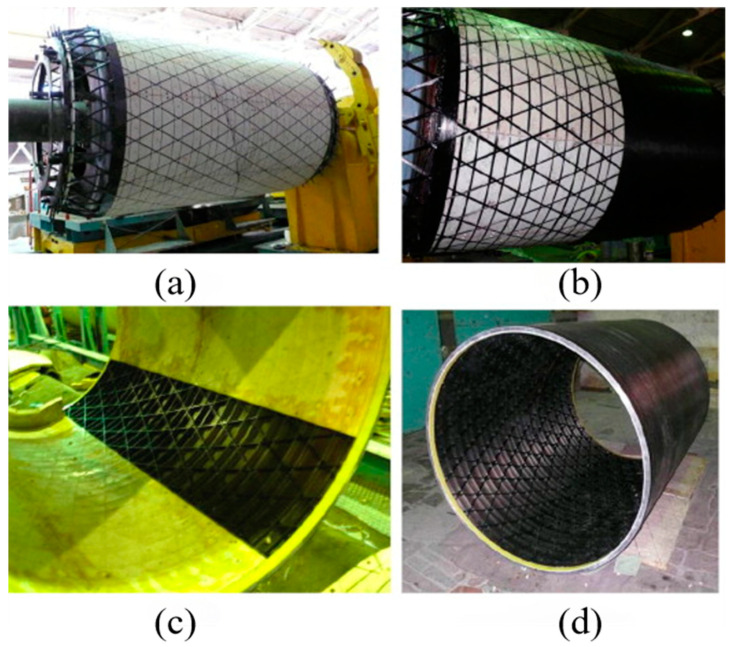
Fabrication of a composite lattice structure: (**a**) winding of the ribs, (**b**) winding of the skin, (**c**) removal of elastic coating, (**d**) fabricated structure. Reproduced from Ref. [124], Copyright Elsevier, 2012.

AFW, with its high level of automation, optimizes anisotropic fiber characteristics and has become a cost-effective technology for composite manufacturing. By continuously winding fiber tows onto a mandrel, it precisely positions fibers and improves structural efficiency through the effective use of high-strength fibers to suit stress paths [122]. AFW is especially suitable for making regular cylindrical composite structures, such as various lightweight shells in the aerospace field, and mass production can significantly reduce costs [123]. Vasiliev’s article documents CRISM’s use of carbon and aramid epoxy composites in filament winding to create high-performance grid structures for various aerospace applications, such as rocket motor casings and aircraft components. CRISM’s process for manufacturing skinned and un-skinned grid structures involves elastic coatings with grooves formed in silicone rubber [124]. The sequential fabrication steps are illustrated in Figure 5.

**Figure 6:**
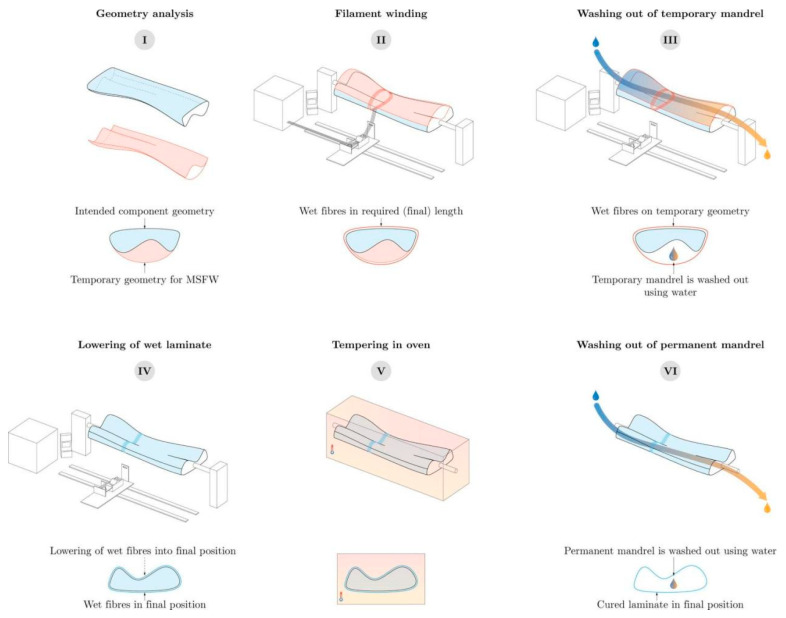
Schematic sequence of multi-stage filament winding. Reproduced from Ref. [126], Copyright Elsevier, 2021.

As technology advances, AFW is evolving towards multi-stage and coreless systems. Since AFW involves winding resin-impregnated tows onto mandrels for rapid and cost-effective manufacturing, most fiber-wound components are rotationally symmetric. Non-axisymmetric geometries are either challenging or impossible to produce [125]. To make more complex structures, multiple mandrels are needed. The multi-stage fiber winding (MSFW) method sustainably produces lightweight fiber-reinforced composites with complex geometries [126]. It uses water-soluble winding mandrels made of sandy composite materials. Reusable materials enable eco-friendly winding in phases, allowing for asymmetric double-curvature and undercut geometries. Figure 6 shows an overview of the MSFW stages.

**Figure 7:**
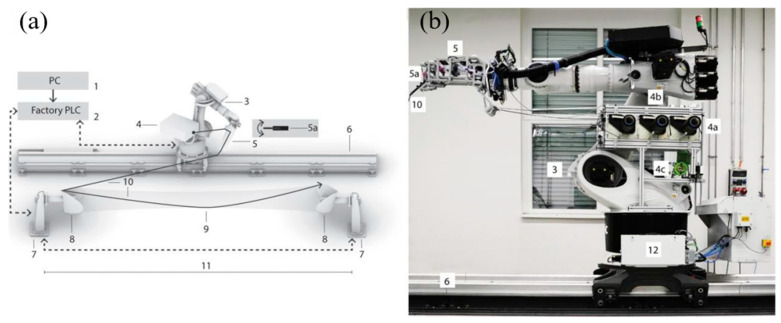
CFW system: (**a**) conceptual diagram; (**b**) implemented system. Components: 1. computer; 2. ICD fabrication laboratory PLC; 3. industrial robot (KR420); 4. fiber guiding and impregnation system: 4a. CF/GF fiber creel, 4b. passive tensioning system (mechanical dancer bar), 4c. peristaltic pump: Albin ALP 09-F connected to Polycarboxylic epoxy resin source; 5. fiber impregnation end-effector; 5a. tension sensor (Tensometric M-1191-KA); 6. linear track, length 10 m; 7. digitally synchronized 1-axis positioners (KP1), no core, or mechanical synchronization needed; 8. modular winding effectors, steel, weight 75 kg; 9. multi-material G/CFRP composite; 10. fiber bundle under pretension; fiber bundle on the composite body; 11. adjustable distance between winding tools allows the AM of any component length in the 1 to 10-m range; 12. BEC Box: digital/analog sensors and actuators integration unit. CF, carbon fiber; CPRCFW, cyber–physical robotic coreless filament winding; G/CFRP, glass/carbon fiber-Reinforced polymer; GF, glass fibers; PLC, programmable logic controller Reproduced from Ref. [129], Mary Ann Liebert, Inc.2022.plc.

Coreless Fiber Winding (CFW) is an innovative robotic manufacturing technique for producing fiber-polymer composite structures. It adds complexity to the collaborative design framework of the composite industry. CFW adapts the AFW process used in the composite industry to manufacture larger and more complex shells and tanks for the aerospace field [127]. CFW differs from AFW in that component shapes are defined by multiple anchor points connected by straight lines in a specific order. Geometric deviations in the fiber network stem from fiber–fiber interactions. In the hybrid form, fibers are partly surface-supported. CFW decouples the tool from the composite structure, cutting tooling costs and boosting component cost-effectiveness. However, it increases fiber–fiber interactions, which are hard to predict and control [128]. The complete CFW system layout is depicted in Figure 7.

It can be seen that conventional AFW can significantly reduce the material waste and cycle time of reducing axisymmetric parts, and non-axisymmetric parts save on tooling costs, although they require expensive multi-axis equipment and centerless equipment, which increases equipment cost expenditures.

#### 4.1.2. Automated Tape Laying (ATL)

Automated Tape Laying (ATL) is one of the most mature automated composite manufacturing technologies and is increasingly utilized in aerospace applications [130]. This process automatically places pre-impregnated fiber tapes (prepreg), typically 25–250 mm wide, along programmed directions to construct large composite structures with customized layups and fiber orientations. ATL systems, commonly mounted on gantry platforms, incorporate material storage, heating zones, consolidation rollers, and peel-ply handling mechanisms. State-of-the-art equipment can process prepregs in varying widths (75, 150, or 300 mm) [131] and operates in two primary configurations: Flat Tape Laydown Machines (FTLM) for planar surfaces and Contour Tape Laydown Machines (CTLM) for gently curved geometries [35]. These gantry-based systems achieve 10-axis motion (5 axes on the gantry and 5 on the placement head), enabling the fabrication of components, such as wing skins with slight contours. ATL is particularly suited for large, flat, or low-curvature aircraft components, including wings (e.g., the F-22), empennage structures (e.g., the Boeing 777), and stabilizers [36]. A key advantage is its compatibility with robotic systems and gantries, facilitating multi-axis maneuverability for large structures [132]. Despite higher deposition rates and lower equipment and material costs compared to automated fiber placement (AFP), due to reduced prepreg slitting and spooling complexity, ATL generates significant waste when producing complex geometries [40]. This limitation necessitates larger runoff areas and scrap courses than AFP methods [132]. In practice, ATL is often combined with secondary processes, like double diaphragm forming, as demonstrated in GKN’s production of Airbus A400M wing spars [40].

#### 4.1.3. Automated Fiber Placement (AFP)

**Figure 8:**
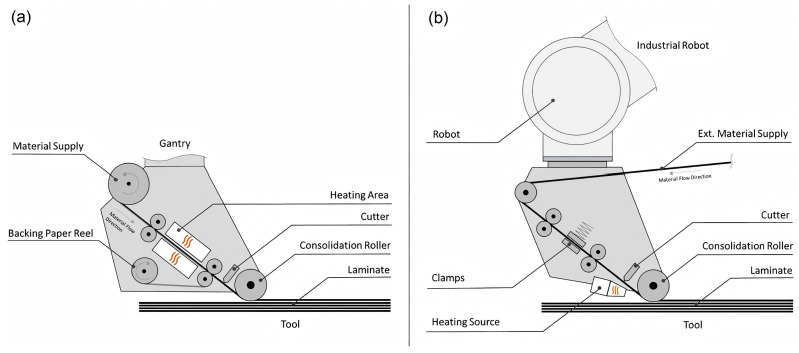
Schematic fiber placement heads: (**a**) ATL, (**b**) AFP. Reproduced from Ref. [136], Elsevier, 2024.

Functioning as a hybrid system that integrates ATL and AFW technologies, AFP utilizes prepreg tows or slit tapes, typically ranging in width from 0.125 to 0.5 inches [38]. Reported labor savings reach 50% for complex, contoured parts manufactured via AFP compared to manual methods [133]. A direct comparison of the placement heads of ATL and AFP is provided in Figure 8. Aerospace manufacturers document overall reductions in person-hours of up to 70–85% and a decrease in scrap rates from 25% to 5% through automation [39]. Typical aerospace applications include wing spars and fuselage sections [134]. Despite these advantages for large-scale aircraft production, the high capital costs of traditional AFP systems hinder widespread adoption [135].

Additionally, however, AFP technology faces significant technological limitations, especially in quality control. At high stacking speeds, insufficient compaction and heating can cause delamination defects due to poor interlaminar bonding and increased void content, ultimately undermining structural integrity [137]. For example, imperfections, such as gaps and overlaps, caused by rapid layup speeds have been shown to propagate delaminations under mechanical loading, thus reducing impact resistance and overall performance [138]. Croft et al. [139] examined the relationship between defects and compression strength, revealing a notable 15–20% decrease in compression strength compared to defect-free baselines, with the strength loss remaining fairly constant for larger gap dimensions. Nik et al. [140] demonstrated that increasing the number of tows while decreasing their width can significantly reduce defect area percentages. For a gap area of approximately 10%, the buckling load reduction varies between 10 and 12%, depending on the specific laminate layup. These challenges highlight that AFP is not a universal solution and requires careful process optimization to reduce defect formation [141]. Effective monitoring of out-of-plane defects demands advanced solutions, as manual inspection accounts for 32–50% of total production time and often fails to meet specification requirements [142].

Defect reduction necessitates breakthroughs spanning detection systems, deposition mechanisms, and process parameter control. Tang et al. [143] developed an inline inspection, which has been proven to locate out-of-plane defects within 1 s, achieving over 72% Intersection over Union (IoU) segmentation accuracy. Separately, addressing layup Pressure Error (LPE)—a critical quality factor—Xu et al. [144] optimized heavy-duty robotic AFP mechanisms by analyzing how robot and end-effector errors affect LPE. This approach reduced the compaction force error by 69.1% and enhanced the uniformity of pressure distribution. Consequently, layup defects decreased by 70.97%, confirming that the optimized robot posture enhances layup pressure uniformity and accuracy, reduces defect occurrence, and improves product quality [144]. Xie et al. [145] presented a control strategy for AFP process parameters to recognize, locate defects, and stabilize layup surface temperature. Infrared thermography, combined with path control, delivered over 94% edge detection on single-ply edges and an interlayer gap measurement of under 10%. Their temperature control model effectively curbed defects caused by thermal fluctuations, demonstrating the effectiveness of infrared thermography in defect detection and reduction for FRP composites.

### 4.2. Curing and Consolidation Innovations (Autoclave, OoA, In Situ)

The curing and consolidation stages are the ones that lock in the composite’s final properties. The goal is to achieve a fully densified part with a minimum of voids, as porosity has a highly detrimental effect on mechanical properties, particularly interlaminar shear and compression strength.

#### 4.2.1. Autoclave Molding

Autoclave molding technology, dating back to the 1940s, was initially used for manufacturing thermosetting composite parts for aircraft primary load-bearing structures. An autoclave is a pressure vessel with a heating system. It is one of the most mature composite-structure molding technologies, accounting for over 80% of aerospace composite production [48]. This technology cures thermosetting resin–matrix composite structures using high-temperature compressed gas within the autoclave. The composite structures feature a high fiber volume content, low porosity, and reliable mechanical properties due to the high temperature and pressure. Zeeshan Ali et al. [146] tested the tensile strength of AFRP cured in autoclaves and those cured without autoclaves. The mechanical properties of aramid fiber-reinforced composites before and after autoclave treatment are shown in Table 6. After autoclave treatment at 100 °C, 140 °C, and 180 °C, tensile strengths of 206.48, 223.07, and 234.79 MPa were obtained. The high tensile strength of 234.79 MPa achieved after autoclaving at 180 °C under constant pressure was 21.70% higher than that of non-autoclave-cured AFRP. M. Akay et al. [147] compared the properties of Kevlar-49/epoxy laminates prepared in autoclaves with those cured in ovens. The oven-cured laminates had fewer voids, resulting in lower water absorption rates and smaller water diffusion coefficients. This effectively alleviated the reduction in glass transition temperature and deterioration of mechanical properties caused by autoclave curing and water absorption.

Autoclave molding technology has long been crucial in producing high-performance composite structures for the aerospace industry, such as composite wings, fuselages, and other load-bearing components. Currently, manufacturing large composite parts requires massive or even supermassive autoclaves to ensure internal part quality, with an increasing trend in autoclave size. Integrated molding technologies for aerospace components, such as these large autoclaves, enable the integrated molding of large composite structures. This significantly reduces the number of parts and fasteners required inside an aircraft, thereby lowering its overall weight. Moreover, blended wing-body configurations made through this technology result in smoother aircraft surfaces. This design not only enhances aerodynamic efficiency but is also easier to implement. For instance, the blended wing-body passenger aircraft developed by Northwestern Polytechnical University shows a remarkable increase in lift-to-drag ratio and noticeably improved aerodynamic performance. Most importantly, the elimination of weak joints boosts the aircraft’s safety.

#### 4.2.2. Out of Autoclave

Out of Autoclave (OoA) technology has emerged as a low-cost alternative to traditional autoclave curing processes, particularly in the aerospace industry [36]. Unlike autoclave curing, which requires maintaining high-temperature and pressure environments for extended periods, OoA involves curing composite structures within a vacuum bag, typically using an oven or heat blankets. This approach eliminates the need for expensive autoclave equipment and significantly reduces energy consumption and production costs. For instance, the MC-21 aircraft, developed by Russia’s Aero Composite, was the first commercial aircraft to utilize OoA technology for manufacturing its composite wings. The manufacturing cost of these wings was only one-seventh of that associated with autoclave molding technology [148].

Despite its cost advantages, OoA faces challenges in achieving the same mechanical performance as autoclave-cured parts. One major issue is the high porosity of composite structures cured under OoA conditions, which can reach certain levels due to the lower pressure environment [50]. To address this, researchers have developed specialized resin systems and process techniques. Centea et al. [149] introduced a resin system designed to efficiently evacuate voids. Additionally, the double-vacuum-bag (DVB) technology, invented by NASA in 2004, has shown promise in reducing porosity and improving fiber volume fraction. This technology utilizes two vacuum bags and a steel diaphragm, applying external atmospheric pressure while maintaining a near-vacuum environment internally during the curing process. Rana et al. [150] demonstrated that DVB technology enhances interlaminar shear strength and flexural strength compared to single-bag methods, attributing the improvements to better volatile management and fiber compaction.

Another challenge is the low fiber volume fraction in OoA-cured composites. Northrop Grumman addressed this issue by developing a fragile, unidirectional prepreg tape specifically for automatic taping, which enables increased fiber content and pressure resistance in composite fuel storage tanks [151]. Furthermore, advancements in OoA prepreg materials have enabled the production of parts with quality nearly equivalent to autoclave-cured parts. These prepregs are designed to be compatible with lower-cost setups, eliminating defects typically associated with autoclave manufacturing. For example, VBO (Vacuum Bag Only) prepregs exhibit breathable properties that enable entrapped gases to migrate and escape during processing, resulting in low-porosity parts. The latest OoA prepregs are being developed to achieve fiber volumes and void contents comparable to those of autoclave-cured parts, even at lower cure pressures [149].

In summary, while OoA technology offers significant cost and energy savings, ongoing research and development continue to enhance its mechanical performance and quality, making it an increasingly viable alternative to traditional autoclave processes in composite manufacturing.

#### 4.2.3. In Situ Consolidation

**Figure 9:**
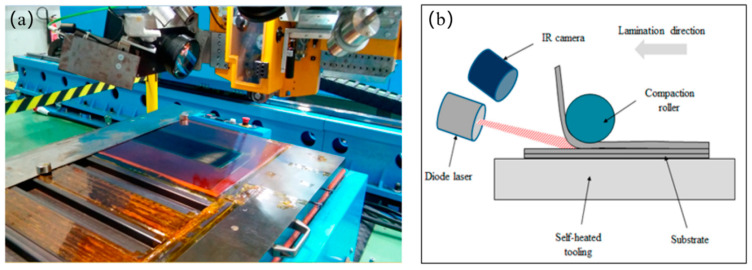
In situ consolidation equipment in FIDAMC (Foundation for the Research, Development and Application of Composite Materials) (**a**); and a schematic diagram of the manufacturing process including manufacturing tooling, compaction roller, a diode laser, and an infrared thermographic camera (**b**). Reproduced from Ref. [153], MDPI, 2020.

In situ consolidation involves rapidly heating the thermoplastic polymer matrix above its melting point during automated layup, enabling the merging of melted surfaces upon cooling under compaction pressure, thereby eliminating the need for secondary curing cycles in autoclaves or ovens [152]. The experimental setup and process schematics are depicted in Figure 9.

The application of in situ consolidation to aerospace primary structures, however, faces challenges. The high melting points and viscosities of high-performance thermoplastic matrices (e.g., PEEK, PEKK) necessitate demanding processing conditions (high temperatures and pressures) for effective consolidation during layup [154]. Furthermore, entrapped air, volatiles, or inadequate processing parameters can lead to increased void content, a critical defect that is detrimental to the mechanical properties of the final component [155]. Controlling void formation and achieving sufficient interlaminar bonding strength are therefore paramount for structural integrity. Process complexity arises from numerous interacting physical parameters, including heating source characteristics, layup speed, compaction force, tooling temperature, and substrate preheating [156]. These parameters significantly influence the degree of intimate contact, polymer chain diffusion, cooling rates, residual stress development, and ultimately, porosity levels and interlaminar bond quality [157]. Optimizing these parameters for high layup speeds is essential for industrial viability.

Significant hardware development has focused on placement heads to enhance process control and consolidation quality [152]. Patents filed by major aerospace entities, like Boeing (1997, 2002) and Aerospatiale/Airbus (2003, 2011), and machine manufacturers, like Cincinnati Milacron and Automated Dynamics, detail advancements in heating methods, compaction systems, tooling solutions, and process control for automated thermoplastic layup with in situ consolidation [153]. Parameter optimization is essential for achieving high-quality in situ consolidation without secondary steps. Wong et al. [158] studied the tow-winding in situ consolidation of aramid fiber-reinforced nylon 6 and achieved complete consolidation with just 0.25% porosity, matching the quality of compression-molded parts. This is crucial for aerospace components, which have strict void content limits [154].

The economic viability of thermoplastic composites in aerospace depends on balancing material costs and manufacturing savings. Cost–benefit analyses suggest that in situ consolidation eliminates autoclaving and enables high integration, offering significant cost advantages over autoclave-cured thermosets and offsetting the initial material premium [159,160]. Projects, like MULTIFAL in Clean Sky 2 and the ALCAS wing box demonstrator, aim to harness the cost and productivity benefits of in situ consolidation, such as lower cycle times, reduced energy consumption, and integrated manufacturing, to make thermoplastic composites economically viable for large-scale aerospace applications [161]. Thus, in situ consolidation is key to unlocking the potential of thermoplastic composites in next-generation, cost-effective aerospace manufacturing.

### 4.3. Additive Manufacturing of AFRP (3D/4D Printing): Potential and Hurdles

Three-dimensional printing is a process of “connecting materials to manufacture objects based on 3D model data, usually in a layer-by-layer manner” [162]. Compared to ATL, 3D printing can produce complex composite structures without the typical waste associated with traditional manufacturing methods. The dimensions and geometry of composites can be precisely controlled with computer-aided design. Thus, 3D printing of composites combines process flexibility with the creation of high-performance products.

**Figure 10:**
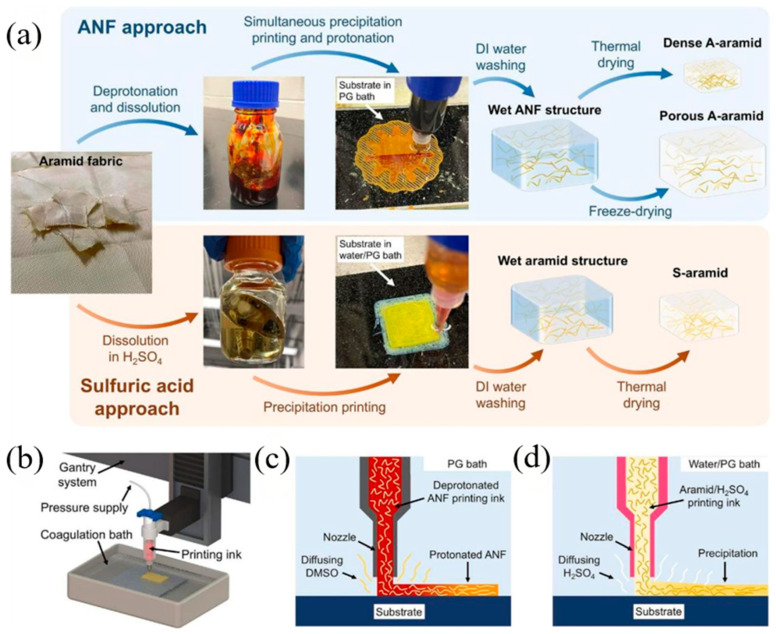
(**a**) Flowchart of the additive manufacturing processes of all-aramid materials through the ANF approach and sulfuric acid approach. (**b**) Precipitation printing setup. (**c**) Printing mechanism illustration of the ANF approach. (**d**) Printing mechanism illustration of the sulfuric acid approach. Reproduced from Ref. [165], Copyright Elsevier, 2025.

FDM is the most commonly used 3D-printing technology for AFRP. FDM creates 3D objects by heating thermoplastic materials to a molten state and then depositing them layer by layer through a nozzle. Its key advantages are high efficiency and cost-effectiveness, making it ideal for small-batch production and prototyping. In aerospace, FDM is often used to manufacture lightweight structural components and complex parts. Kuchampudi Sandeep Varma et al. [163] used aramid fiber-reinforced PETG (PETG-KF) as FDM printing material. They combined a back-propagation neural network (BPNN) model to understand the nonlinear relationship between input parameters and mechanical properties, and used ANOVA to evaluate the significance of the parameters. This provided a structured method to boost the mechanical performance of 3D-printed objects. Wang et al. [164] explored enhancing the strength and ductility of short-carbon-fiber and Kevlar-reinforced ABS-based composites simultaneously via FDM. The results showed that under optimized 3D printing conditions, short carbon and Kevlar fibers can effectively reinforce ABS-based composites, achieving a balance between flexural strength and ductility. The study by Tu et al. [165] shows that the dense all-aramid structures produced by the ANF method exhibited excellent mechanical properties, with a Young’s modulus of 7.2 GPa and a tensile strength of 146.6 MPa. The structures are also able to withstand extreme environments, including temperatures up to 350 °C. As a result, high-performance all-aramid 3D structures can be realized through ANF-based precipitation 3D printing and can be used as lightweight structures or thermal protection components in aircraft systems. The complete additive-manufacturing workflow and precipitation-printing setup are summarized in Figure 10.

SLA, a photo-polymerization method that utilizes acrylic-based resins with photoinitiators and laser activation, offers advantages, such as producing high-quality objects with resolutions as low as 10 microns and smooth surfaces. Sachini D. Perera et al. [166] added ANFs to photoresist and used SLA to print ANF-nanocomposites. Defect-free parts were achieved with the addition of 1.50 wt% ANF. ANFs enhanced the mechanical properties of photoresists as nanoscale reinforcing agents without affecting printability. However, SLA resins require liquid monomers or oligomers for rapid light-induced polymerization, but often fail to meet industrial manufacturing requirements due to inadequate thermal and mechanical properties, as well as formulation challenges.

Despite the advantages mentioned above, practical additive manufacturing of AFRP remains limited by low fiber volume fractions—currently below 30 wt% for FDM and 15 wt% for SLA—and by significant anisotropy: in-plane modulus can be 3–7 times higher than transverse values, while inter-layer porosities of 5–20% further weaken out-of-plane properties [167]. As a result, 3D-printed AFRP parts usually have 40–60% lower off-axis strength than autoclave-cured laminates, restricting their use to secondary or non-load-bearing aerospace applications until these challenges are addressed [168].

**Figure 11:**
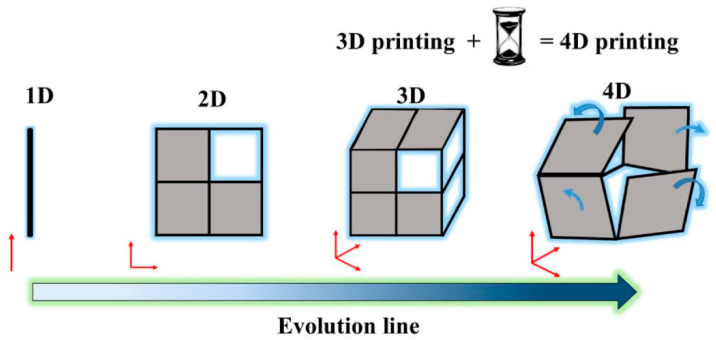
Illustration of the evolution of 1D to 4D printing over time. Four-dimensional printing is the addition of a time dimension to 3D printing. Reproduced from Ref. [169], Copyright Elsevier, 2023.

Four-dimensional printing, as shown in Figure 11 [169], involves adding a fourth dimension, time, to 3D printing. The differences between 3D printing and 4D printing are shown in Table 7. Smart materials are essential for 4D printing, as they enable printed structures to undergo self-transformation in physical properties and functionality over time when exposed to specific environmental stimuli. SMPCs stand out due to their low density, cost, diverse types, and excellent designability, making 4D printing with SMPCs a favorite among researchers [170,171,172].

The applications of 4D printing in the aerospace industry can be divided into three levels: components, mechanisms, and structures. At the component level, actuators can replace traditional mechanical structures by utilizing components created through 4D printing technology [169].

Dong et al. [173] proposed an innovative strategy for printing continuous-fiber-reinforced porous composites. They fabricated porous composite structures using a PLA matrix and Kevlar fibers to study the effects of printing and structural parameters on mechanical and shape-memory properties. The mechanical and shape-memory properties of lightweight structures can be designed and optimized by adjusting relevant printing parameters and fiber content. Wang et al. [174] presented a direct-ink-writing-based 4D printing method for AFRP. Continuous aramid fibers can support free-standing structures during printing, improving the mechanical and deformation properties of 4D-printed structures. The printed liquid-crystal aramid composites could withstand loads up to 2805 times their weight and achieved a bending deformation curvature of 0.33 mm^−1^ at 150 °C. While still in its early stages, 4D printing holds immense potential for creating self-deploying, adaptive, and morphing aerospace structures, fundamentally changing the way components are designed and utilized.

### 4.4. Processing and Assembly of AFRP Structures

Successful fabrication of an AFRP component by molding or autolay is only half the battle; next, the component must be precision machined to meet final geometric tolerances and assembled into a larger, functional aerospace structure. This final processing and assembly phase is fraught with unique challenges that stem directly from the inherent properties of AFs. The high toughness of the AFRP material not only makes it excellent in terms of impact resistance but also makes it difficult to avoid damage when cutting and drilling. Similarly, the chosen joining method has a profound effect on the weight, integrity, and long-term reliability of the final structure. This section will focus on the state-of-the-art technologies and strategies currently employed to overcome these critical post-fabrication challenges.

#### 4.4.1. Cutting

Unlike brittle materials, like carbon fiber composites, aramid fibers tend to bend and deform under a cutting edge rather than fracturing cleanly. This behavior, rooted in the fiber’s high toughness and low transverse shear strength, is the primary cause of common machining defects, such as extensive burr formation, fiber pull-out, and delamination, all of which can severely compromise the component’s surface quality, dimensional accuracy, and structural integrity [20,175].

To mitigate these issues, research has focused on optimizing both conventional and non-traditional machining processes:

Studies have shown that cutting parameters, such as spindle speed, feed rate, cutting width, and vibration amplitude, significantly influence the cutting force and surface quality of AFRP. Higher spindle speeds and vibration amplitudes tend to reduce burr formation and cutting forces by enhancing the shearing action and promoting smoother fiber fracture. For example, Mughal et al. [175] found that increasing the spindle speed from 500 to 5000 rpm reduced burr height by 60.82% and 71.00% for plane and toothed disc cutters, respectively. Similarly, Wen et al. [176] demonstrated that cutting at a speed of 91.11 m/s (spindle speed of 30,000 rpm) with a cutting depth of 0.2 mm and a feed speed of 5 mm/s could achieve a surface roughness (Ra) as low as 32 nm. This indicates that optimizing cutting parameters is crucial for improving the surface quality of AFRP.

Tool design also plays a vital role in minimizing cutting defects. Specialized tools, such as diamond-coated cutters and ultrasonic-assisted tools, have been developed to enhance cutting performance. Diamond-coated tools, for instance, offer better wear resistance and sharpness, which help in reducing cutting forces and improving surface finish. Ultrasonic-assisted machining, on the other hand, introduces high-frequency vibrations to the cutting tool, which reduces friction between the tool and workpiece, breaks chips effectively, and decreases tool wear. This technique has been found to be particularly effective in machining brittle and hard materials, such as AFRP. Research has shown that ultrasonic-assisted machining can significantly reduce cutting forces and improve surface quality compared to conventional machining [177].

Non-traditional machining techniques, such as laser cutting, water jet cutting, and abrasive water jet cutting, have also been investigated for their potential to improve the cutting quality of AFRP. Laser cutting, which relies on thermal energy to vaporize material, offers advantages, like minimal material waste and smooth edge cuts. However, it can cause thermal damage to the resin matrix, resulting in reduced mechanical properties [178]. Water jet cutting, especially abrasive water jet cutting, is advantageous due to its low heat input, which minimizes thermal deformation and delamination. It also produces less dust and noise compared to conventional methods. Nevertheless, it suffers from limitations, such as high noise levels and the inability to machine blind holes. Abrasive water jet cutting, in particular, has been found to be more feasible for AFRP due to its material removal mechanism, higher removal rates, and superior surface finish [179].

The formation mechanism of burrs during cutting has been analyzed through fly-cutting experiments and mechanical modeling. Bao et al. [20] established a cutting mechanical model to predict the effects of the angle between the feed direction and fiber orientation on tensile and shear behavior during cutting. Their results indicated that tensile forces form significant burr defects during the cutting process, while fewer burrs are observed when the fibers are under shear stress. The study also revealed that increasing cutting speed and tool sharpness could reduce burr formation. Furthermore, the elastic recovery phenomenon, which occurs with increasing cutting depth, has a significant impact on machining quality.

In summary, the cutting of AFRP in aerospace applications requires a careful balance of machining parameters, tool design, and process selection to minimize defects and enhance surface quality. While traditional cutting methods remain widely used due to their availability and lower capital costs, non-traditional techniques offer promising solutions for improving the machinability of AFRP. Future research should focus on developing hybrid machining processes that combine the advantages of both traditional and non-traditional methods, as well as optimizing cutting tools and parameters for specific applications of AFRP.

#### 4.4.2. Drilling

The processing and assembly of AFRP structures often involve drilling operations, which present unique challenges due to the material’s anisotropic and heterogeneous nature. Drilling is a critical process that can significantly affect the mechanical performance and fatigue life of AFRP components. The drilling process for AFRP is complex and can lead to various defects, including delamination, fiber pull-out, and resin burn, which compromise the composite’s structural integrity [180]. Delamination is particularly problematic as it can drastically reduce the mechanical performance of the drilled components, making it a primary concern in the manufacturing process.

To address these challenges, researchers have investigated the impact of various drilling parameters and tool geometries on the drilling process. The selection of optimal drilling parameters, including feed rate, spindle speed, and drill diameter, is crucial for minimizing delamination and other drilling-induced defects. For instance, studies have shown that lower feed rates and higher spindle speeds can help reduce delamination by reducing the thrust force exerted on the material [181]. Additionally, the use of specialized drill geometries, such as step drills and brad-point drills, is effective in reducing delamination and improving hole quality [182].

The tool wear during drilling also significantly impacts the quality of the drilled holes. Carbide drills have been found to outperform high-speed steel (HSS) drills in terms of tool life and delamination control [183]. Coated tools, such as those with TiN or AlCrN coatings, further enhance performance by reducing friction and improving wear resistance [184]. However, the choice of tool material and geometry must be carefully considered based on the specific requirements of the application and the characteristics of the AFRP material being processed.

Recent advancements in drilling techniques for AFRP include the use of minimum quantity lubrication (MQL) and cryogenic drilling. MQL has been shown to reduce cutting forces and improve surface quality by minimizing heat generation and material deformation [185]. Cryogenic drilling, on the other hand, involves the use of liquid nitrogen or other cryogenic coolants to lower the cutting temperature, thereby reducing thermal damage and tool wear. This technique has proven effective in enhancing the machinability of AFRP and producing high-quality holes with minimal delamination [186].

Despite these advancements, further research is still needed to optimize drilling processes for AFRP. Future work should focus on developing more sophisticated analytical and simulation models to predict and control delamination during drilling. Additionally, the integration of real-time monitoring and adaptive control systems could further improve the efficiency and reliability of drilling operations for AFRP structures in aerospace applications.

#### 4.4.3. Joining

Joining individual AFRP components into a cohesive and reliable aerospace structure is a critical final step. The choice of joining method—primarily mechanical fastening or adhesive bonding—directly impacts the structure’s weight, load-bearing capacity, fatigue life, and inspectability. Adhesive bonding has gained prominence due to its ability to provide a continuous connection, which enhances the structural integrity and load-bearing capacity of AFRP components. As highlighted by Vinson [187], adhesive bonding can achieve high strength-to-weight ratios and improved fatigue resistance compared to mechanical fastening. This is attributed to the uniform stress distribution and the absence of stress concentrations that are typical in mechanically fastened joints. Moreover, the use of adhesives can also offer benefits, such as improved aerodynamic smoothness and enhanced visual appearance, which are crucial for aerospace applications. The fatigue life of adhesively bonded AFRP structures can be significantly longer than that of riveted or spot-welded structures, with some studies reporting fatigue lives twenty times longer [187].

However, adhesive bonding is not without its challenges. The process is susceptible to surface preparation, environmental conditions, and curing parameters. For instance, poor surface preparation or contamination can lead to adhesive failure, characterized by the absence of adhesion on one or both bonding surfaces [188]. Additionally, the presence of defects, such as porosity, voids, and disbands, can significantly compromise the performance of adhesively bonded joints. These defects can arise from manufacturing uncertainties and operational environmental factors, making the prediction and detection of such defects a complex task [188].

Mechanical fastening, on the other hand, offers ease of disassembly and inspection, making it a preferred method for specific aerospace applications. It involves the use of bolts, rivets, or other fasteners to join AFRP components. This method is beneficial when components need to be accessed for maintenance or repair. However, mechanical fastening introduces stress concentrations around the fastener holes, which can lead to reduced structural integrity and potential failure points. Drilling holes for fasteners can also damage the reinforcing fibers, thereby affecting the mechanical properties of the composite [189].

To address the limitations of individual joining methods, hybrid joining techniques that combine adhesive bonding and mechanical fastening have been explored. These hybrid joints aim to leverage the advantages of both approaches while mitigating their disadvantages. For example, the use of adhesive in conjunction with bolts or rivets can provide enhanced load-bearing capacity and fatigue resistance. Studies have shown that hybrid joints can exhibit superior mechanical performance compared to purely adhesive or purely mechanical joints [190].

The selection of an appropriate joining method depends on several factors, including the specific requirements of the aerospace application, the material properties of the AFRP components, and the manufacturing and operational constraints. In some cases, the use of secondary mechanical fasteners in adhesively bonded joints is mandated by certification authorities to ensure safety and reliability. This conservative approach, however, can lead to increased weight and manufacturing complexity [191].

In conclusion, the joining of AFRP structures in aerospace applications requires careful consideration of the available joining techniques. While adhesive bonding offers significant benefits in terms of structural performance and weight reduction, it is essential to address the associated challenges related to defect formation and uncertainty quantification. Mechanical fastening provides ease of disassembly and inspection but introduces stress concentrations. Hybrid joining techniques present a promising solution by combining the strengths of both methods. Future research should focus on developing advanced inspection techniques and probabilistic reliability-based design optimization frameworks to enhance confidence in the use of adhesively bonded joints in critical aerospace applications.

### 4.5. Surface Quality Control and Metrology for Aerospace-Grade Components

In addition to achieving the macroscopic geometry and internal properties of components, ensuring high-quality surface micro-geometry is equally crucial for aerospace applications. Surface quality—including roughness, waviness, and defects—directly impacts aerodynamic performance, coating adhesion, fatigue life, and assembly precision. Consequently, precise control and quantitative characterization of surface quality throughout the manufacturing process are key to guaranteeing the final performance and reliability of AFRP components.

#### 4.5.1. Key Surface Quality Metrics and Their Manufacturing Origins

The surface quality of aerospace components is typically defined by a series of critical metrics. Surface roughness (such as Ra, Rz) describes the microscopic smoothness of a surface, directly influencing aerodynamic drag and the physical bonding strength between coatings and substrates [178]. Waviness indicates more macroscopic, periodic surface undulations, largely affecting assembly precision and contact stress distribution between components. Form/profile tolerances measure the conformity of the actual component surface to its nominal design model. Typical surface defects also include print-through from the curing process, resin-rich/poor areas, as well as scratches and burrs introduced during machining [192].

These surface quality metrics are closely tied to manufacturing processes. For instance, in automated fiber placement (AFP), factors such as the curvature of layup paths, the precision of tow overlaps/gaps, and the uniformity of the compaction roller pressure directly determine the final surface waviness and defect density [193]. Uneven temperature or pressure during curing can lead to insufficient resin flow, resulting in resin-rich regions or print-through of fibers. As discussed in Section 4.4, the high toughness of aramid fibers makes them particularly prone to burrs and tearing during machining, severely compromising surface quality [194].

#### 4.5.2. Advanced Metrology and Applications

The precise quantitative evaluation of the above-mentioned surface quality metrics requires advanced metrology techniques.

For macroscopic geometric features—such as waviness and form/profile tolerances—non-contact three-dimensional optical measurement technologies, like laser profilometry and structured light scanning, have become standard tools. These methods enable rapid and highly accurate acquisition of complete surface 3D point cloud data, allowing quantification of surface undulations caused by processes, such as AFP. Studies have shown that 3D laser scanning can accurately correlate the wavelength and amplitude of surface waviness (e.g., wavelengths as small as 0.1–0.2 mm in regions of tight curvature) with specific AFP process parameters, thereby facilitating process optimization and online quality monitoring [195].

For assessing microscopic surface features and damage, higher-resolution detection methods are required. Optical and confocal microscopy are frequently used to observe and quantify fine scratches and burr morphologies on machined surfaces. Furthermore, to reveal subsurface damage potentially induced during manufacturing, X-ray computed tomography (X-ray CT) offers irreplaceable capabilities [196]. For example, in the drilling of aramid composites, CT scanning has been applied to non-destructively reconstruct the three-dimensional morphology of subsurface damage around hole inlets, accurately quantifying visually undetectable entry tears and exit delamination. It has been demonstrated that the use of specially designed drill bits can reduce the damaged area by approximately 40% [197].

Moreover, surface quality assessment extends beyond geometric characteristics to include functional properties—particularly for components requiring coatings. Atomic force microscopy (AFM) and contact angle goniometry are key tools for characterizing surface functionality. AFM enables quantification of nanoscale roughness and surface energy, while contact angle measurements assess wettability [198]. Studies have demonstrated that plasma treatment can substantially increase the surface energy of AFRPMC substrates (e.g., from 40 mJ/m^2^ to over 70 mJ/m^2^), which in turn enhances coating adhesion strength by about 60% compared to samples subjected only to mechanical abrasion [199]. This highlights the importance of precise measurement of surface chemical state and energy as a prerequisite for ensuring the long-term reliability of coatings.

In summary, surface quality control of AFRP aerospace components is a systems engineering process spanning the entire manufacturing chain. It not only requires the optimization of forming and machining processes but also depends on advanced metrology techniques for comprehensive and precise characterization—from macro-level geometric profiles to a microstructural surface morphology and chemical state. Such rigorous quality control is fundamental to achieving the design performance and high reliability required for aerospace-grade AFRP components.

## 5. Aerospace Applications of AFRP: Case Studies and Performance Evaluation

Building on the fundamental properties and manufacturing techniques discussed in the previous sections, this section provides an in-depth analysis of AFRP’s performance in real-world aerospace applications. The value of the material is not only in its data sheet properties but also in its performance, reliability, and cost-effectiveness in complex engineered systems. Through a series of carefully selected case studies, this section will analyze the successes and limitations of AFRP in detail, directly addressing the fundamental challenges of these materials in terms of compressive strength, interfacial bonding, and environmental durability. This analysis aims to provide a comprehensive understanding, revealing the strengths of AFRP, as well as the significant barriers it faces, and exploring how its unique benefits can be maximized through engineering solutions.

### 5.1. Case Studies

#### 5.1.1. Flight Service Evaluation of AFRP Panels in Commercial Aircraft

The decade-long in-flight service evaluation project, led by NASA on the Kevlar-49/epoxy composite secondary structural components (such as wing-body fairings, engine aft fairings) of the Lockheed L-1011 TriStar wide-body passenger aircraft, has offered a wealth of empirical data on the long-term performance of AFRP in real aviation settings [200,201].

The evaluation indicates that these AFRP components performed well over a decade of service. There were no major structural issues affecting flight safety, nor were there any significant corrective measures needed. The primary defects observed were minor impact damage from ground operations or foreign object strikes during flight (like surface scratches and small dents), minor wear and elongation around fastener holes, and localized adhesion loss or delamination over small areas. The type and severity of these defects are essentially comparable to those observed in glass-fiber-reinforced composite components commonly used in aircraft [202].

A parallel study exposed Kevlar-49/epoxy composite specimens to outdoor environments in different parts of the US for up to three years. Their residual mechanical properties remained above 80% of the original, indicating good environmental durability [203].

Though the Lockheed L-1011 TriStar was not a commercial success, the project robustly demonstrated the long-term reliability and economic viability of AFRP in civil aircraft secondary structures. It laid the foundation for the subsequent use of AFRP by Airbus and Boeing in building lightweight, high-strength components for their passenger aircraft to enhance fuel efficiency and cut operational costs [202,204]. 

#### 5.1.2. AFRP Application in Space Self-Lubricating Components or Hypervelocity Impact Shielding Systems

AFRP demonstrates unique potential in addressing the specialized functional needs of space. In the field of space self-lubrication, Colas et al. [205] primarily evaluated the tribological performance of PTFE-based composites filled with MoS_2_, glass, and mineral fibers in both vacuum and atmospheric environments. They found that the PTFE/(10% MoS_2_, 25% glass fiber) composition exhibits good transfer film-forming ability and a stable friction coefficient [206]. However, aramid fibers have a low friction coefficient and good wear resistance [207]. This makes them a strong candidate for developing high-performance space self-lubricating composites (such as those for bearings and bushings), which could extend the in-orbit life and reliability of space mechanisms.

In terms of spacecraft high-velocity impact protection, Nam et al. [208] proposed a multi-functional protective structure based on aramid/epoxy composites. This structure not only exhibits excellent protective performance, comparable to that of unmodified original aramid/epoxy composites, when withstanding projectile impacts at speeds of 2.7–3.2 km/s but also achieves microwave absorption of −10 dB in the frequency range of 6.65–18 GHz, thereby imparting specific stealth characteristics to the spacecraft [206].

This research highlights the potential of AFRP in integrating structural protection with electromagnetic functions, offering new ways to integrate highly lightweight spacecraft systems. However, for self-lubricating applications, more long-term performance data on aramid-based self-lubricating composites in real space environments is needed. As for multi-functional protective systems, further evaluation of the stability of their multi-functional coupling performance in complex space environments (such as atomic oxygen and charged particle radiation) is required.

#### 5.1.3. Compression and Compression-After-Impact Performance of Kevlar-49/Epoxy Composites in UAV Structures

A study on the structural application potential of UAVs focused on evaluating the compressive and CAI properties of Kevlar-49/epoxy composites with a 12-layer [0/90/±45/0/90]s symmetric and balanced layup and a fiber volume fraction of approximately 41% [206]. The results clearly showed that impact damage (mainly internal delamination) significantly reduced the remaining compressive strength of Kevlar-49/epoxy composites. The higher the impact energy, the larger the internal delamination area, and the lower the CAI strength. The primary reason for the strength reduction is that the impact-induced internal delamination of the laminate causes local buckling of the sub-laminate under subsequent compressive loads, thereby triggering premature structural instability and failure [209].

The study also pointed out that to achieve better CAI strength, the outermost layers of the laminate should be fiber-laid in the 0° direction (i.e., parallel to the main compressive load direction). This helps suppress surface damage growth and enhances the structure’s anti-buckling ability [209].

This case highlights the inherent limitations of AFRP in applications dominated by compressive loads or those requiring significant compressive loads after impact. For example, in the main load-bearing structures of UAVs, AFRP has a relatively low compressive strength and a substantial reduction in remaining compressive strength after exposure to effects [18]. This means that in designing such structures, more cautious damage-tolerant design standards must be adopted. It may be necessary to set lower design allowable strain values or compensate for material deficiencies through structural optimization and precise damage assessment to ensure structural safety.

### 5.2. Performance Evaluation and Analysis

A comprehensive evaluation of the performance of AFRP in aeronautical and aerospace applications requires a thorough assessment of their behavior in specific working environments, their durability over extended service periods, and their unique damage and failure mechanisms.

AFRP demonstrates excellent adaptability in extreme temperature conditions. At cryogenic temperatures, aramid fibers exhibit minimal brittleness and strength loss. For instance, at a liquid nitrogen temperature (−196 °C or −320 °F), the strength loss is extremely small, making them highly suitable for space components that come into contact with cryogenic propellants [210]. At high temperatures, while aramid fibers have a carbonization temperature of approximately 427 °C (800 °F), the maximum service temperature of AFRP is typically constrained by the thermal stability of the polymer matrix, which is usually around 150–177 °C [18]. Notably, aramid fibers exhibit a slight negative coefficient of thermal expansion in the axial direction (e.g., −2.4 × 10^−6^/°C for Hexcel aramid fibers), which is crucial for manufacturing precision components with high dimensional stability, such as satellite antenna reflectors, that operate in environments with severe in-orbit temperature fluctuations [211].

In the harsh space environment, the performance of AFRP is challenged by various factors, including vacuum, ultraviolet (UV) radiation, high-energy charged particles, and atomic oxygen. High vacuum conditions can lead to outgassing in some polymer matrices, which may contaminate sensitive instruments. Therefore, it is necessary to select low-outgassing resins and conduct relevant tests under ultrahigh vacuum (UHV) conditions. Aramid fibers are relatively sensitive to UV radiation, and prolonged exposure can lead to degradation in performance. Studies have shown that the use of coatings embedded with nanoparticles can effectively provide protection, reducing the degradation of Kevlar fabrics after one week of UV exposure to 5% of the original level [212]. In terms of vibration and acoustic environments, AFRP generally exhibits superior damping characteristics compared to metallic materials, enabling effective dissipation of vibration energy [213]. For example, the use of aramid layers in the winglets of the Airbus A320 aircraft is reported to reduce wear on fasteners by 15%, partly due to the vibration-damping properties of aramid [214]. In terms of antennas, Zhang et al. [215] developed an ultra-lightweight Kevlar/polyimide 3D woven spacer microstrip antenna (3DWS-MA-T), which achieved excellent electromagnetic performance (gain value > 5 dB) and good impedance matching (S11 value < −20 dB), as well as an operating frequency close to the designed frequency of 2.4 GHz. In addition, the antenna exhibited perfect structural integrity, maintaining appropriate resonant frequency and impedance matching after experiencing an 8 J impact or exposure to extremely low/high temperatures. These characteristics help to enhance the fatigue life and stability of structural components, making it easy to integrate into aircrafts or satellites.

The long-term performance and durability of AFRP are crucial for its successful application in the aeronautical and aerospace fields. The decade-long flight service evaluation of L-1011 aircraft components has confirmed their reliability in secondary structures when properly designed and maintained. However, moisture absorption is a key issue that requires attention in AFRP. Aramid fibers have a hygroscopic tendency, and water absorption can lead not only to fiber swelling and a decrease in mechanical properties, particularly compressive strength, but also to the plasticization of the polymer matrix. This significantly reduces its glass transition temperature (Tg), thereby affecting the high-temperature performance and stiffness of the composite [216]. Additionally, under sustained loads, aramid fibers exhibit a certain degree of creep behavior, which can increase the Young’s modulus [217]. Therefore, it is essential to fully account for performance reductions in wet and hot environments during design and application and to establish practical inspection and maintenance strategies. Non-destructive testing (NDT) techniques, such as ultrasonic C-scanning, can be utilized to detect potential damage [218].

Understanding the damage mechanisms and failure modes of AFRP is vital for its safe design. Under tensile loads, AFRP typically exhibits progressive fiber fracture, with high toughness and fracture energy [219]. However, their compressive strength is relatively low, and their failure modes are complex, often involving micro-buckling of fibers, kinking, and delamination. Under impact loads, AFRP absorbs a significant amount of energy through various mechanisms, including fiber fracture, matrix cracking, interfacial debonding, and inter-laminar delamination, demonstrating excellent impact resistance. Nevertheless, the internal damage formed after impact, particularly delamination, can severely weaken their residual compressive strength. Regarding fatigue, the S-N curve of Kevlar/914 (epoxy) composites shows that their fatigue life is highly sensitive to the applied stress amplitude and stress ratio (R-value) [220]. The presence of harmful stress components (compression) can significantly degrade fatigue performance and alter the failure mode from fiber fracture to more complex mechanisms such as matrix cracking, delamination, and fiber wear. A prominent advantage of AFRP is its “non-catastrophic” failure behavior. They tend to absorb energy by generating extensive micro-damage, with relatively slow crack propagation and often obvious precursors before final failure. This provides valuable damage-tolerance and fail-safe characteristics for structures.

## 6. Challenges, Innovative Solutions, and Future Perspectives for AFRP Applications

As this review demonstrates, AFRP occupies an indispensable position in aerospace engineering due to its unique low density and high toughness. However, several significant and interrelated challenges need to be overcome to realize the full potential of these materials. At the end of this section, we will summarize the key bottlenecks discussed in this paper, assess the most promising new solutions, and develop a strategic roadmap for future research and development. The goal of this study is to provide a clear perspective that helps the scientific community overcome existing barriers and propel AFRP into a new era of advanced, smart, and sustainable aerospace applications, drawing on the insights from recent comprehensive analyses.

### 6.1. Overarching Technical Challenges

**Figure 12:**
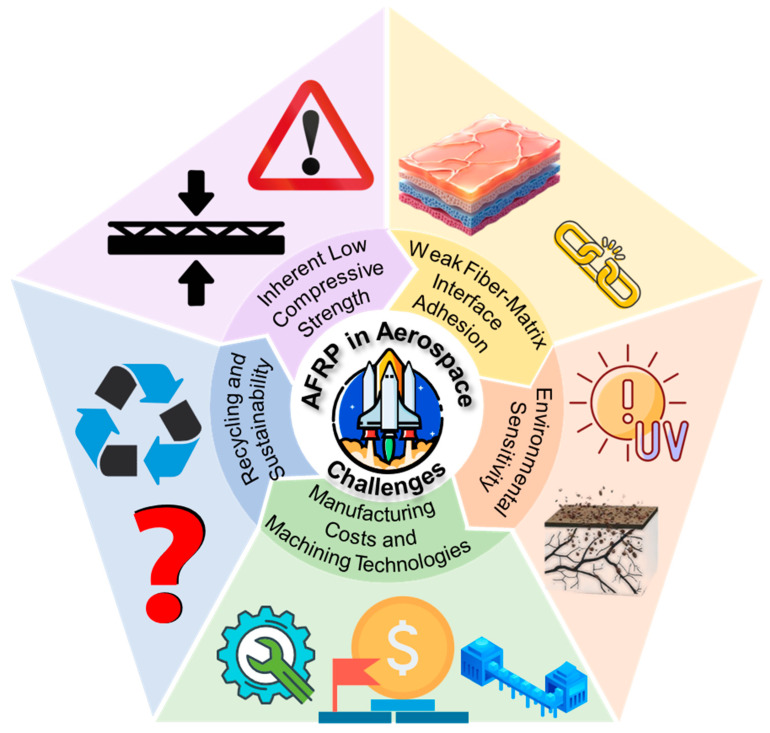
Overarching technical challenges.

A confluence of interconnected technical hurdles fundamentally constrains the widespread adoption and performance maximization of AFRP. For a consolidated overview of these challenges, see Figure 12. The most significant of these is the material’s inherent low compressive strength, often a mere 15–20% of its tensile capability, which is a direct consequence of the micro-buckling failure mode of the rigid-rod aramid fibers [221]. This weakness not only curtails their use in primary load-bearing structures but also results in poor compression-after-impact (CAI) performance, a critical metric for damage-tolerant design [209]. This primary mechanical deficiency is exacerbated by the weak fiber–matrix interface adhesion, stemming from the chemically inert and smooth surface of aramid fibers, which impedes efficient stress transfer and compromises properties, such as interlaminar shear strength [17].

Furthermore, AFRP exhibits a pronounced sensitivity to the aerospace operational environment. Their susceptibility to moisture absorption can lead to matrix plasticization and dimensional instability, while long-term UV exposure can cause photochemical degradation, both of which threaten their long-term durability [15]. Finally, barriers to broader adoption are presented by practical and economic factors, including high manufacturing costs and the notorious difficulty of machining these rigid composites without inducing defects [19]. In an increasingly environmentally conscious industry, the challenge of recycling and sustainability for these thermoset-based materials also looms large, demanding lifecycle-oriented solutions [221,222].

### 6.2. Emerging Innovative Solutions and Breakthroughs

**Figure 13:**
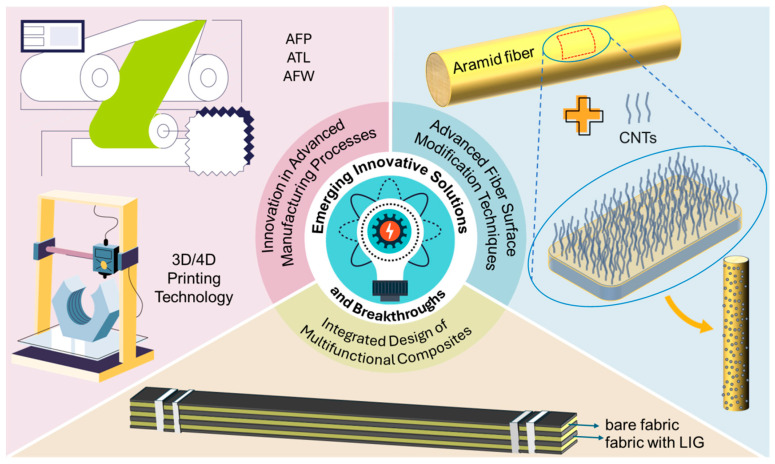
Emerging innovative solutions and breakthroughs.

In response to these challenges, the research landscape is vibrant with innovative solutions that are beginning to shift the performance paradigm for AFRP. For a consolidated overview of these emerging solutions, see Figure 13. To combat the critical challenge of weak interfacial adhesion, the most pivotal strategy lies in advanced fiber surface modification and interfacial engineering. By creating multi-scale, functional interphases through techniques, like plasma treatment, bio-inspired polydopamine (PDA) coatings, or the growth of nanostructures, like CNTs, researchers have demonstrated dramatic improvements in interfacial shear strength—in some cases by over 60%—directly enhancing the composite’s overall robustness and, by extension, its compressive performance [59].

Simultaneously, a paradigm shift is underway, moving from viewing AFRP as simple structural materials to designing integrated multifunctional composites. By embedding capabilities, such as strain sensing for structural health monitoring or incorporating materials for electromagnetic wave absorption, their value proposition is significantly enhanced, enabling system-level benefits beyond mere weight reduction [206]. Innovations across the materials and manufacturing spectrum critically enable this evolution. The exploration of high-performance thermoplastic matrices, such as PEEK, promises improved toughness and recyclability [223]. The application of Computational Materials Engineering (CME) is accelerating the design-test-analysis cycle for these complex material systems [224]. Crucially, these material-level innovations are being brought to life by advancements in manufacturing processes, where technologies, like Automated Fiber Placement (AFP) and additive manufacturing, are making the production of complex, high-quality AFRP components more efficient and economically viable.

### 6.3. Future Research Directions and Roadmap

**Figure 14:**
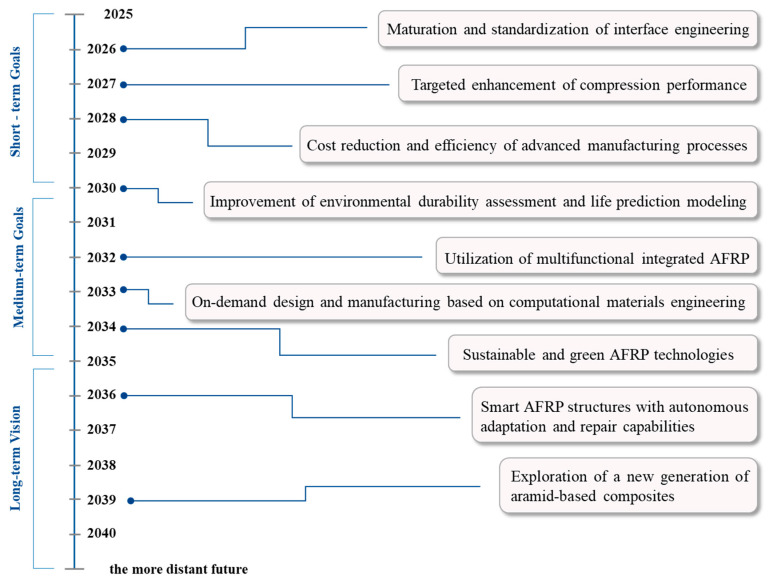
Future research directions and roadmap.

To fully harness the potential of AFRP and systematically address the remaining challenges, future research should follow a strategic roadmap, progressing from near-term optimization to long-term disruptive innovation. For a consolidated overview of these future research directions, see Figure 14.

#### 6.3.1. Short-Term Goals (Next~5 Years): Technological Optimization and Deepening of Engineering Applications

**(1)**  **Maturation and standardization of interface engineering.**

The focus is on breaking through the large-scale application of several highly efficient and stable surface modification technologies for aramid fibers, which exhibit good process compatibility (e.g., multilayer self-assembly based on PDA, and low-temperature plasma grafting of specific functional groups), and establishing the corresponding quality control standards. The goal is to stabilize the interfacial shear strength of typical AFRP systems by more than 50%.

**(2)**  **Targeted enhancement of compression performance.**

Combined with optimized interfacial engineering, the mechanisms and effects of fiber pre-stressing, microstructure modulation (e.g., introduction of a small amount of high-modulus nanofillers for synergistic reinforcement), and hybrid design (e.g., synergistic layup with a small amount of high-compression-strength carbon fibers) on the enhancement of compression performance are systematically investigated.

**(3)**  **Cost reduction and efficiency of advanced manufacturing processes.**

Optimize automated layup process parameters to reduce material waste; develop low-damage, high-efficiency cutting and machining technologies and tools for the characteristics of aramid fibers; and promote near-net-shaping technology to minimize the need for secondary processing.

**(4)**  **Improvement of environmental durability assessment and life prediction modeling.**

Establish a more accurate long-term performance evolution database and accelerated aging test method for AFRP in aerospace complex service environments (coupled effects of humidity and heat, UV, temperature cycling, fluid erosion, etc.). Develop life prediction models based on damage mechanics and multi-physical field coupling.

#### 6.3.2. Medium-Term Goals (Next~10–15 Years): Multi-Functional Integration and Intelligent Breakthroughs

**(1)**  **Utilization of multifunctional integrated AFRP.**

Realize efficient integration and reliable application of structural health monitoring (e.g., strain/damage self-awareness network based on piezoresistive effect), electromagnetic shielding/wave absorption, active/passive vibration control, thermal management, etc., in AFRP components. Focusing on the compatibility and durability of the interface between the functional layer and the structural layer.

**(2)**  **On-demand design and manufacturing based on computational materials engineering.**

Construct a multi-scale computational model across the “molecular–microscopic–fine-macroscopic” scale to realize accurate prediction of AFRP material properties, processing, and service behavior. Develop a material reverse design platform based on artificial intelligence and machine learning.

**(3)**  **Sustainable and green AFRP technologies.**

Focus on the development of AFRP systems based on bio-based and recyclable thermoplastic resins; explore efficient and low-cost technologies for recycling and reuse of aramid fibers (e.g., chemical recycling, pyrolysis); and evaluate the performance and application potential of recycled fibers.

#### 6.3.3. Long-Term Vision (>15 Years): Disruptive Innovation and Intelligent Material Systems

**(1)**  **Smart AFRP structures with autonomous adaptation and repair capabilities.**

Explore the integration of smart elements, such as shape memory alloys and polymers, self-repairing microcapsules, and microvascular networks, with AFRP to give the materials the ability to autonomously adjust their shape, stiffness, or repair damage when the external environment changes or damage occurs.

**(2)**  **Exploration of a new generation of aramid-based composites.**

Based on a deeper understanding of the chemical structure and condensed matter physics of aromatic polyamides, we are exploring the next generation of aramid fibers and their composites with new molecular designs, ultra-high mechanical properties (e.g., tensile and compressive properties close to theoretical strengths), extreme environmental resistance, or unique functionalities.

Through continuous investment and collaborative innovation in the above directions, AFRP will surely show broader prospects in responding to the increasingly stringent performance requirements in the aerospace field and expanding brand-new application scenarios, thus contributing to the core material power for the construction of future aerospace equipment that is lighter, faster, stronger, smarter, and more sustainable.

## 7. Conclusions

This review provides an in-depth analysis of the advantages, core challenges, and future directions of AFRP for aerospace applications, conducted through a systematic assessment of its fundamental properties, performance enhancement strategies, advanced manufacturing technologies, and typical application cases. The following main conclusions are obtained:

(1) The value of AFRP in aerospace applications stems from their excellent specific strength and impact toughness. Still, their wider application is limited by two major bottlenecks, namely inherent low compressive strength and weak fiber/matrix interfacial bonding.

(2) The most effective way to enhance the overall performance of composites is to improve interfacial bonding. Surface modification techniques, such as plasma treatment and chemical grafting, have been experimentally demonstrated to improve interfacial strength by more than 50%.

(3) Advanced manufacturing technologies, such as automated layup (AFP), ex situ curing (OoA), and additive manufacturing, are the key to solving the problems of high cost and complex processing of AFRP, which provides the possibility of manufacturing complex high-performance components.

(4) The successful application of AFRP is highly dependent on the structural design of ‘building on strengths and avoiding weaknesses’, i.e., maximizing their strengths in tensile and impact-dominated scenarios and avoiding their shortcomings in compression applications through hybrid design or innovative structural forms.

(5) The future development trend of this material is evolving from a single structural load-bearing to a ‘smart’ material system that integrates sensing, self-healing, shape memory, etc., which is the key to enhancing its core competitiveness in the next generation of aerospace vehicles.

## Figures and Tables

**Table 1:** Detailed comparison of mechanical, thermal, and economic characteristics of AFRP, CFRP, and GFRP.

Property	AFRP	CFRP	GFRP	Reference
Density (g/cm^3^)	1.38–1.47	1.75–2.00	2.50–2.60	[2,4]
Tensile Strength (GPa)	3.0–3.6	3.5–7.0	2.0–3.5	[2,5]
Specific Modulus (GPa/g/cm^3^)	85–120	200–350	35–50	[2,8]
Charpy Impact Strength (kJ/m^2^)	63–80	24	45–50	[3,4]
Glass Transition Temperature (°C)	135–160	160	110–130	[3,6]
Residual Strength Decline after 200 °C Exposure (%)	≈35	≈45	≈50–60	[5,6]
Cost per kg (USD)	80–120	120–250	25–45	[4,7]
Cost Efficiency (Normalized to CFRP, %)	77.3 (hybrids)	100	13.3	[4,7]

**Table 2:** Comparison of properties of meta-Aramid and para-Aramid fibers [22].

Properties	Meta-Aramid (m-AF)	Para-Aramid (p-AF)
Density	~1.38 g/cm	1.43–1.47 g/cm
Molecular Weight	>20,000	>20,000
Mechanical Strength	Relatively lower	Breaking strength of Kevlar: 24.86 cN/dtex, Kevlar-149 modulus: ~1000 cN/dtex
Thermal Stability	Tg: 270 °C, Td: ~450 °C	Tg: 345 °C, Td: ~550 °C
Chemical Inertia	Chemically inert surface	Chemically inert surface
Hygroscopicity	e.g., Nomex moisture regain: 4.5%	e.g., Kevlar-29 moisture regain: 7.0%, Kevlar-49: 3.5–4.5%, Kevlar-149: 1–3%
Drawbacks	Limited axial stretchability, low shear modulus, chemically inert surface, UV degradation	Limited axial stretchability, low shear modulus, chemically inert surface, UV degradation

**Table 4:** Comparison of interfacial engineering techniques for aramid fibers.

Technique	Mechanism	Key Advantages	Key Challenges	Performance Impact	References
Plasma Treatment	Surface activation, polar group introduction, roughness increase	Dry process; High efficiency; Multiple gas options	Surface damage risk; Uniformity issues; Scale-up challenges	ILSS improvement: +45.5%, Surface active groups: +82.4%	[52,53]
Chemical Etching	Surface roughening through chemical reagents	Simple process; Effective roughening	Harsh chemicals; Environmental concerns; Safety issues	Bundle strength: +10.2%	[54]
Chemical Grafting	Covalent bonding of functional molecules	Strong chemical bonds; No tensile strength loss	Multi-step process; Costly; Validation needed	Interfacial shear strength: +51.03%	[55]
Bio-Inspired Coatings	Mussel-inspired adhesion; Hierarchical nanostructure	Mild processing; Environmentally friendly; High adhesion	Complex fabrication; Scale-up challenges	Interfacial adhesion: +43.1%	[55]
Hybrid Sizing	Multi-scale structure with nanoparticles	Excellent interfacial bonding; Multi-functional; High performance	High material cost; Dispersion challenges; Scale-up issues	Flexural strength: +132.6%; Flexural modulus: +99%	[56]

**Table 5:** Composite material preforming methods and aerospace applications.

Preforming Method	Technical Characteristics	Aerospace Application Scenarios
Automated Fiber Winding (AFW)	CNC-controlled winding of dry fibers/prepreg; suitable for axisymmetric structures.	Rocket motor casings, fuel tanks, high-pressure pipelines (e.g., Ceres-1 rocket fairings).
Automated Tape Laying (ATL)	Automated placement of prepreg tapes; ideal for large flat or low-curvature components.	Aircraft wing skins, fuselage panels (e.g., Boeing 787 fuselage sections).
Automated Fiber Placement (AFP)	Combines winding and tape-laying for complex curved surfaces.	Aircraft inlets, fuselage curved structures (e.g., F-35 vertical tail).

**Table 6:** Mechanical properties of aramid fiber-reinforced composites before and after autoclave treatment. Reproduced from Ref. [146], John Wiley and Sons, 2023.

Composite (Configuration)	Heat Treatment (°C)	Time (min)	Tensile Strength (MPa)	Young’s Modulus (GPa)	Work of Fracture (kJ/m^2^)
Aramid fiber	OT	N/A	192.91 ± 7.04	6.29 ± 0.17	15.93 ± 1.31
Aramid fiber	100	25	206.48 ± 6.79	6.69 ± 0.18	16.47 ± 1.18
Aramid fiber	140	25	223.07 ± 2.51	8.68 ± 0.09	4.48 ± 0.70
Aramid fiber	180	25	234.79 ± 2.89	9.02 ± 0.13	4.34 ± 0.51

**Table 7:** Differences between 3D and 4D printing technologies.

Characteristic	3D Printing	4D Printing
Technical Principle	Builds objects layer by layer based on a pre-designed digital model	Based on 3D printing technology with an added time dimension
Curing Method	Requires external energy (such as heat or light) for curing	After curing, the printed object can deform autonomously under specific conditions
Material Requirements	Uses conventional thermoplastic or thermosetting materials	Uses smart materials (such as shape-memory polymers)
Application Fields	Used for manufacturing static objects with complex geometries	Used for manufacturing dynamic objects that can change over time or respond to the environment
Development Stage	Widely used in aerospace, medical, automotive, and other fields	Still in the early stages of development with limited application cases
Time Dimension	No time-dimension variation	Includes a time dimension, allowing printed objects to change over time
